# The synthetic lethality of targeting cell cycle checkpoints and PARPs in cancer treatment

**DOI:** 10.1186/s13045-022-01360-x

**Published:** 2022-10-17

**Authors:** Shuangying Li, Liangliang Wang, Yuanyuan Wang, Changyi Zhang, Zhenya Hong, Zhiqiang Han

**Affiliations:** 1 Department of Obstetrics and Gynecology, Tongji Hospital, Tongji Medical College, Huazhong University of Science and Technology, Wuhan, 430030 Hubei China; 2 Department of Hematology, Tongji Hospital, Tongji Medical College, Huazhong University of Science and Technology, Wuhan, 430030 Hubei China

**Keywords:** Cell cycle checkpoint, PARP inhibitors, Drug resistance, Synthetic lethality, Targeted therapy, Cancer

## Abstract

Continuous cell division is a hallmark of cancer, and the underlying mechanism is tumor genomics instability. Cell cycle checkpoints are critical for enabling an orderly cell cycle and maintaining genome stability during cell division. Based on their distinct functions in cell cycle control, cell cycle checkpoints are classified into two groups: DNA damage checkpoints and DNA replication stress checkpoints. The DNA damage checkpoints (ATM-CHK2-p53) primarily monitor genetic errors and arrest cell cycle progression to facilitate DNA repair. Unfortunately, genes involved in DNA damage checkpoints are frequently mutated in human malignancies. In contrast, genes associated with DNA replication stress checkpoints (ATR-CHK1-WEE1) are rarely mutated in tumors, and cancer cells are highly dependent on these genes to prevent replication catastrophe and secure genome integrity. At present, poly (ADP-ribose) polymerase inhibitors (PARPi) operate through “synthetic lethality” mechanism with mutant DNA repair pathways genes in cancer cells. However, an increasing number of patients are acquiring PARP inhibitor resistance after prolonged treatment. Recent work suggests that a combination therapy of targeting cell cycle checkpoints and PARPs act synergistically to increase the number of DNA errors, compromise the DNA repair machinery, and disrupt the cell cycle, thereby increasing the death rate of cancer cells with DNA repair deficiency or PARP inhibitor resistance. We highlight a combinational strategy involving PARP inhibitors and inhibition of two major cell cycle checkpoint pathways, ATM-CHK2-TP53 and ATR-CHK1-WEE1. The biological functions, resistance mechanisms against PARP inhibitors, advances in preclinical research, and clinical trials are also reviewed.

## Introduction

An intact genome is important to maintain fidelity during DNA duplication and cell division, which are key for the survival of parental and daughter cells. An estimated 70,000 DNA lesions occur in a single human cell each day [1, 2]. DNA can suffer from external assaults and endogenous mutations at any point and any time. External damages can be caused by exposure to ultraviolet (UV) light, X-rays, and anticancer chemicals. The causes of endogenous damages include reactive oxygen species (ROS) activity, base substitutions, spontaneous deamination, chromatin rearrangement, and DNA replication errors [1, 3, 4]. DNA is so precious, and therefore organisms from prokaryotes to eukaryotes have evolved distinct repair mechanisms to cope with various types of damages. These mechanisms are collectively called the DNA damage response (DDR). The 2015 Nobel Prize in Chemistry was awarded to Tomas Lindahl, Paul Modrich, and Aziz Sancar for their pioneering studies on DNA repair mechanisms involving base excision repair (BER), mismatch repair (MMR), and nucleotide excision repair (NER) [5]. Unresolved DNA single-strand breaks (SSBs) frequently translate into more hazardous DNA double-strand breaks (DSBs) during replication, which are repaired mainly by high-fidelity homologous recombination (HR) or error-prone nonhomologous end joining (NHEJ). BRCA1/2 proteins participate mainly in the HR repair (HRR) pathway, and mutations in their genes are associated with elevated susceptibility to the development of ovarian cancer (40–60% and 11–30% for BRCA1and BRCA2, respectively), breast cancer (72% and 69%, respectively; especially BRCA1 (17q21) and BRCA2 (13q13)), prostate cancer (1% and 11.4% for BRCA1 and BRCA2, respectively) and pancreatic cancer (uncertain rate, BRCA2 > BRCA1) [6–8].

To date, 17 proteins have been identified in the PARP superfamily, and most have been classified into four subfamilies: DNA-dependent PARPs (including PARP1, PARP2, and PARP3); tankyrases (namely tankyrase 1 and tankyrase 2); Cys-Cys-Cys-His (CCCH) zinc finger PARPs (including PARP7, PARP12, PARP13.1, and PARP13.2); and macro-PARPs (comprising PARP9, PARP14, and PARP15) based on their functional and structural distinctions. The other members are either much more specific or not typical [9–11]. The 17 members of PARP family share a conserved catalytic domain and only these 3 DNA-dependent PARP members possess Trp-Gly-Arg (WGR) domain which is responsible for DNA interaction in DNA damage response pathway [9]. Both PARP1 and PARP2 catalyze poly(ADP)-ribosylation (PARylation), while PARP3 catalyzes mono (ADP)-ribosylation [9]. So far, the available PARP inhibitors primarily target both PARP1 and PARP2 and the two proteins play an important role in DDR [10]. Considering PARP1 synthesizes nearly 80% PAR chains in DNA repair pathways for mammalian cells among PARP1 and PARP2, this review specifically refers to PARP1 when we talk about “PARP” proteins in the following sections [11]. Recently, six PARP inhibitors (olaparib, rucaparib, niraparib, talazoparib, fluzoparib, and pamiparib) were approved for use in the treatment of ovarian, breast, and pancreatic cancers. PARP inhibitors induce “synthetic lethality,” a mechanism introduced by geneticists a century ago whereby although one of two gene defects exerts little effect on cell survival, both mutations create a synergistic effect that leads to cell death [12]. PARP1 takes a major part in the repair of SSBs, and PARPi cause unrepaired SBSs left. During the process of DNA replication, these SSBs encounter replication forks and tend to collapse them, causing deleterious DSBs. Homologous recombination deficiency (HRD) cells lack high-fidelity HR pathway for DSB repair, and therefore, PARPi are thought to be capable of inducing their death [12]. However, the wide application of PARPi in a clinical setting may lead to resistance against PARPi, and several mechanisms of resistance against PARPi have been identified and shown to be involved in processes such as the restoration of HR and protection of replication forks [13, 14].

Due to the prevalence of various types of DNA damage, orderly cell cycle progression is critical for cell duplication. Attenuation of DNA replication fine-tuning, which is mainly due to replication stress (RS), causes cell cycle arrest at cell cycle checkpoints that facilitates repair mechanisms. Recent studies have found that G1-S transition points generally becomes nonfunctional in cancer cells, rather than subsequent cell cycle progression [15, 16]. The G1-S transition is controlled by cell cycle checkpoints ATM/CHK2/p53 signals and the interplay between cyclin-dependent kinases (CDKs), CDK inhibitors (CKIs), and the anaphase-promoting complex/cyclosome (APC/C). Normally, cells enter temporary quiescence, senescence, or even nonreversible cell death in the pre-replicative G1 phase in the presence of irreparable DNA damage via p53-mediated pathways or through other gene defects besides p53 mutations. However, common mutations of p53 in cancers impair cell cycle exit [15]. Moreover, CDK2 (mainly CDK2: CyclinE1), its inhibitor p27Kip1, and APC/C inhibitor Emi proteins were found to manipulate the switching, timing, and irreversible transition at the G1 and S phases, respectively. The dysfunction of such regulators stimulates transcription factor E2F, which promotes the commitment entry into the S phase [17]. Normal cells have two major cell cycle checkpoint-associated pathways, ATM/CHK2/p53 and ATR/CHK1/WEE1, which are involved in the surveillance of the G1/S and G2/M checkpoints. Based on their distinct functions in cell cycle control, cell cycle checkpoints are classified into two groups: DNA damage checkpoints (ATM/CHK2/p53) and DNA replication stress checkpoints (ATR/CHK1/WEE1) [15]. Interestingly, members of the former signaling pathway are frequently mutated across a broad spectrum of cancer types, but genomic mutations in the latter pathway are rare [15]. Presumably, cancer cells exploit alterations of ATM/CHK2/p53to avoid cell cycle exit at the G1 phase and rely on ATR/CHK1/WEE1 activity to handle a high level of RS during continuous cell division. From a cell cycle control perspective, mutations in the ATM/CHK2/p53 signaling pathway play a role in promoting cancers, while the inhibition of ATR/CHK1/WEE1 pathway activation produces an antitumor effect. Moreover, these mechanisms synergize with the action of PARPi to some extent, as shown in preclinical researches and clinical trials.

What are the differences between synthetic lethality mechanisms associated with distinct cell cycle checkpoints and PARP inhibitors? Collectively, cell cycle checkpoints and PARPs interact, with certain functions overlapping, in either the RS response or DDR, creating an Achilles’ heel in tumor treatment. Therefore, we briefly review the causes of the DDR and RS response, the molecular features of cell cycle checkpoints and PARP1, several mainstream PARP inhibitor resistance mechanisms, and the latest preclinical and clinical trials enrolling combinations of cell cycle checkpoint agents and PARPi in cancer therapy, especially for PARP-inhibitor-resistant cancers, which are currently used in clinical settings.

## Causes of DNA damage response and replication stress response

**Fig. 1:**
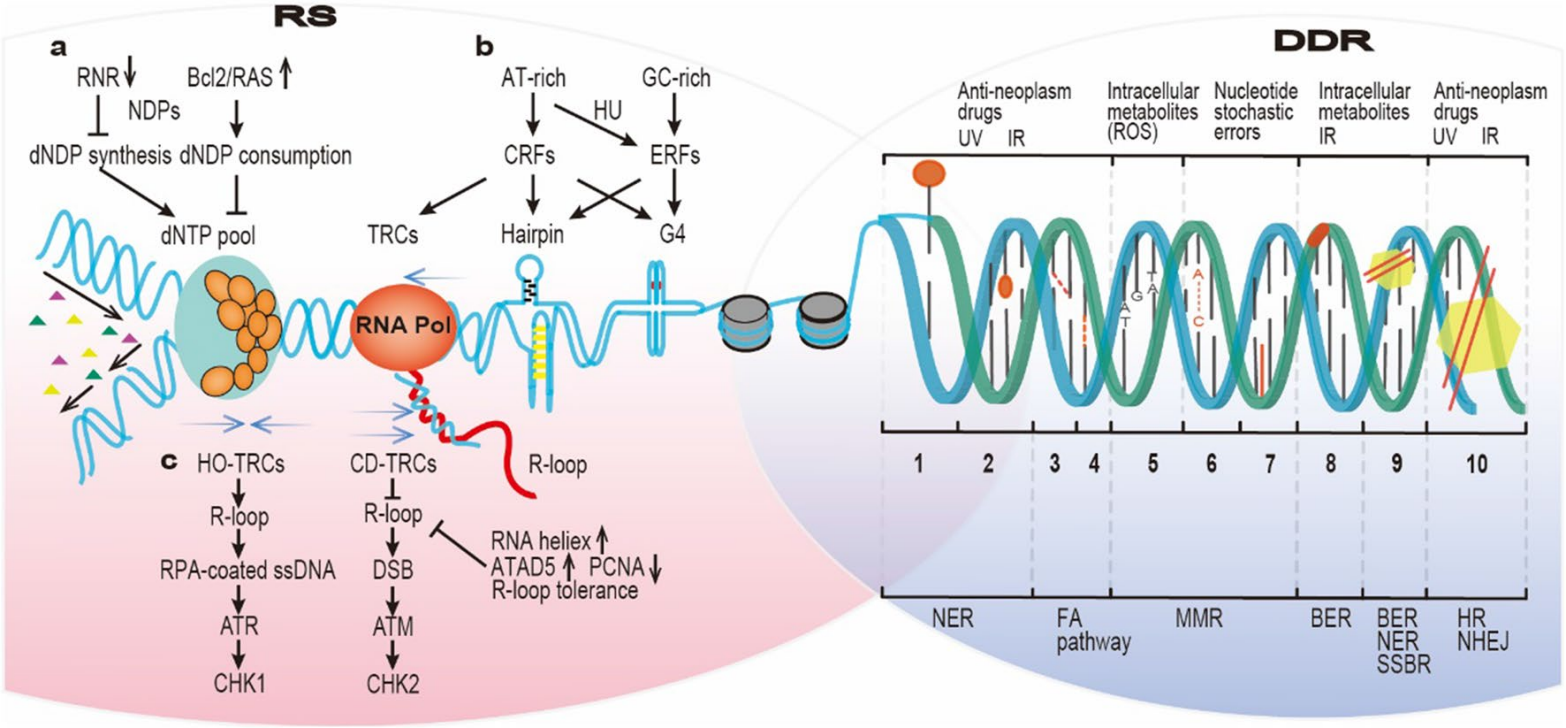
Causes of the DNA damage response and replication stress response. The progression of replication forks faces frequent obstacles, such as RS due to various factors, including **a** reduced dNTPs pool, **b** repetitive sequence-composed DNA fragile sites, **c** TRCs and associated R-loop formation. Additionally, DNA undergoes constantly intrinsic and extrinsic assaults leading to the DDR. Intrinsic assaults include disabled DNA repair, nucleotide stochastic errors, and intracellular metabolite (e.g. ROS) activity, while extrinsic assaults include UV, IR, and anticancer drugs. Several bulky DNA adducts, such as DNA‒protein adducts, DNA intrastrand cross-links and DNA interstrand cross-links, are common causes of DDR and RS. The numbers on the right side represent: 1. DNA‒protein adducts, 2. bulky DNA adducts, 3. DNA intrastrand cross-links, 4. DNA interstrand crosslinks, 5. Base deletion, 6. DNA mismatches, 7. Base insertion, 8. Abasic sites, 9. Single-strand DNA breaks, 10. Double-strand DNA breaks. *dNTPs* Deoxyribonucleoside triphosphates; *TRCs* Transcription–replication conflicts; *ROS* Reactive oxygen species; *UV* Ultraviolet; *IR* Ionizing radiation; *NER* Nucleotide excision repair; *FA pathway* Fanconi anemia pathway; *MMR* Mismatch repair; *BER* Base excision repair; *SSBR* Single-strand break repair; *HR* Homologous recombination; *NHEJ* Nonhomologous end joining

Cell genome stability is constantly under threat by both endogenous factors and exogenous assaults. In addition, DNA replication frequently undergoes disruption for various reasons. Some causes of the DNA damage response (DDR) or replication stress response (RSR) are independent, while others are shared as shown in Fig. 1.

### The primary causes of the DDR

Approximately 55,000 SSBs have been estimated to occur in each cell every day, accounting for more than three-quarters of all DNA lesions. Moreover, although the incidence of DSBs is approximately 25-fold less than that of SSBs per one cell every day, DSBs are much more deleterious to cells [1]. In defense of genetic integrity, distinct cell cycle checkpoints, DNA repair pathways, and even apoptosis ensure the survival of cells. An intrinsic deficiency in DNA repair capability is the leading cause of DNA damage in certain cancers. For example, hereditary mutations of the BRCA1/BRCA2 genes in breast or ovarian cancer disable high-fidelity HRR of harmful DSBs [18]. In cancer cells, stochastic errors arise between purine or pyrimidine nucleotides, which are mediated by DNA polymerases. It was posited that in TP53 codon areas, *C* > *T* transitions at the CpG island occupy approximately one-quarter of overall mutations [19]. Furthermore, intracellular metabolites, ROS, which are produced during normal oxidative respiration, continuous redox reactions, and even immune responses against inflection or inflammation, can elicit SSBs and abasic site mutations or other forms of base damage [20]. Moreover, DNA molecular stability is constantly subjected to UV light exposure from the sun, which causes the largest portion of exogenous damages and is deleterious to genome stability. Primarily, DNA bases, as chromophores, can absorb UVA light, which activates ROS production [21]. CPD (CPD photolyase) and 6–4 photoproducts [(6–4) photolyase] are the major cytotoxic lesions caused by UV radiation and are classified as helix-distorting lesions, strictly speaking. They can be repaired via NER pathway [22, 23]. Other environmental threats include ionizing radiation such as X-rays, which are associated with DSB formation and subsequent repair via HRR or NHEJ pathway [20]. Additionally, most antineoplastic chemicals predominantly target nuclear DNA, causing catastrophic DNA damage. For example, in long-term chemotherapy, platinum binds to purine bases at the N7 position to form platinum–DNA crosslinks, most of which are intra-crosslinks found on the same single strand of DNA [24].

### The causes that trigger replication stress

#### Defective dNTP pool

For mammalian cells that enter the S phase, a balanced and readily accessible pool of four deoxyribonucleoside triphosphates (dNTPs) is a requirement to ensure DNA replication. Disruption of nucleotide metabolism or deficiency in the dNTP pool mitigates fork progression, giving rise to the accumulation of single-stranded DNA (ssDNA) which in turn causes high levels of RS and, sometimes, genome instability. The dominant factors that cause dNTPs shortage are synthesis deficiency or overconsumption. The rate-limiting step for dNTPs synthesis is the transformation of RNA building blocks, that is, nucleotide diphosphates (NDPs), into DNA building blocks, that is, deoxyribonucleotide diphosphates (dNDPs), a reduction reaction catalyzed by ribonucleotide reductase (RNR) [25]. Two catalytic subunits (RRM1), and two regulatory subunits (RRM2 and RRM2B), constitute RNR, a tetrameric enzyme, with RRM2 being the rate-limiting factor for catalytic activity [4]. The overexpression of oncogenes BCL2 and RAS reduces RRM2 activity twofold by binding to it or by modulating the transcription of E2F4, which compromises the activity of RNR and leads to defective dNTPs. Furthermore, cyclinE or human papillomavirus (HPV) E6/E7 stimulates sustained cell proliferation and consumption of dNTPs. Another oncogene, c-MYC, plays the opposite role, accelerating dNTPs biosynthesis. Cyclin E activates CDK2 to drive S and HPV inactivates p53 and pRb that control G1/S transition leading to increased dNTPs consumption. While another oncogene, c-MYC, as a transcription factor, activates genes involved in the biosynthesis of dNTPs to promote S phase and, in general, increases the level of dNTP pool [4, 26]. Overall, excessive dNTPs consumption or defective RNR activity leads to a signaling cascade that results in RS during DNA synthesis [3].

#### AT- or GC-enriched fragile hubs

Repetitive DNA sequences, such as poly (dA:dT) tracts or CpG-enriched dinucleotides cause replication fork stalling/collapse, in addition to nucleotide shortages. These repetitive sequences preferentially develop at early replicating fragile sites and late-replicating common fragile sites (ERFSs and CFSs, respectively). The genetic sequences of the former are controversial. In 2014, Barlow et al. suggested that ERFSs are enriched with GC dinucleotides, while Tubbs et al. indicated that poly(dA:dT) tracts were observed in ERFSs after low hydroxyurea (HU) treatment [27, 28]. Functionally, ERFSs are the replication origin and are transcribed when they enrich in genes, which indicates that they prematurely consumed dNTPs storage and increased collisions between replication and transcription, both of which are causes of RS [28]. In stark contrast to ERFSs, CFSs reduce fork speed, causing a delay at replication origins or a transcription–replication conflict (TRC) owing to their co-occurrence involving very large genes, and inducing viral integration, cancer-related stress, and genomic disorder [29]. These fragile sites are sources of common forms of fragility. The scarcity of protection provided by replication protein A (RPA) and the formation of a non-B secondary structure (e.g. triplex, cruciform, hairpin, and G-quadruplex) hinder the progression of replication and induce breakage due to stress, sometimes spontaneously [25, 27]. In addition to topological torsion, G-4 motifs, stacking tetrads formed by self-recognizing guanines have been associated in several studies with breakpoints, somatic mutagenesis, cancerous gene amplification, and replication forks, which together lead to genome instability [30]. Taken together, these results indicate that due to repeated DNA sequences, some replication sites are fragile and form distinct non-B secondary structures that block replication fork progression [29].

#### Transcription–replication conflicts (TRCs) and R-loops

With inevitable spatial and temporal encounters between RNA polymerase and replisomes, TRCs are found impairing DNA replication [31]. The orientation of TRCs, head-on (HO) or co-directional (CD), has been found to be associated with the frequency of R-loops [32]. An R-loop, which is a tripartite structure composed of a nascent RNA, template DNA strand, and nonhybridized ssDNA, forms more frequently and more astatically because of HO pathway collisions. However, R-loops are resolved in CD TRCs, illustrating that an HO-based TRCs are more detrimental than CD-based TRCs to replication forks [32]. TRCs derived from different pathway collisions cause various types of DNA damage and stimulate distinct RS signaling pathways [32]. HO orientation stimulation of the ATR/CHK1 pathway and associated proteins RPAS33, MUS81, and H2AX have been proven, indicating the formation of ssDNA. However, ssDNA in a replication fork may be either newly generated or a displaced ssDNA sequence [32, 33]. Furthermore, ATM-CHK2 signaling has been found to be activated during CD collisions, and corresponding molecules KAP1, RPAS4/8, and γ-H2AX are involved which indicates that DSBs occur at such sites [32]. In addition to self-resolving R-loops in the regions affected by CD TRCs, cells have evolved various R-loop-unwinding strategies, consisting of accessible R-loop resolution mediated by RNA helicases (DDX1, DDX5, DDX21, and DHX9), and R-loop production-inhibiting strategies, in which ATAD5 (constituting a portion of the replication factor C (RFC)-like complex, RLC) disrupts PCNA (the sliding clamp on replicative polymerases in eukaryotes) loading and R-loop tolerance. Overall, conflicts between replication and transcription pathways induce R-loop formation, causing RS [34].

### The intertwined causes of the RS response and DDR

Reticular cross-links are formed between the sources of RS response and DDR. On one hand, when constant RS is not prevented, the resulting stalled replication forks are inclined to break into DSBs, which generates other types of endogenous DNA damage, including genetic mutations and chromosomal rearrangements. The scarcity of dNTP nucleotides caused by oncogene overexpression leads to an increase in the number of replication origin sites, reduced fork speed, and, ultimately, replication fork collapse, which results in DSB generation. Additionally, unresolved R-loops commonly retain displaced ssDNA sequences, which are substrates for endonucleases that generate DSBs [1]. Alternatively, the untranscribed ssDNA tracts are substrates of APOBEC3, which mediates cytosine deamination, leading to the activation of the BER pathway and inducing DSBs [35]. Similarly, unprocessed RNA‒DNA hybrids arising from R-loop stimulate NER signaling and induce the production of DSBs. In fragile regions, such as ERFs and TRC sites, DNA is prone to suffer from different types of mutations or genome rearrangements [1]. On the other hand, several types of DNA damage are barriers to the expansion of replicative strands. These barriers include bulky DNA adducts, DNA‒protein adducts, and interstrand or intrastrand DNA crosslinks, which are obstacles to DNA replication [25]. Overall, unrepaired replication errors can result in grievous DSBs, and preexisting DNA damage may block replication.

## The biological functions of PARP1 and the ATM/CHK2/TP53, ATR/CHK1/WEE1 pathways in the DDR and RS response

**Fig. 2:**
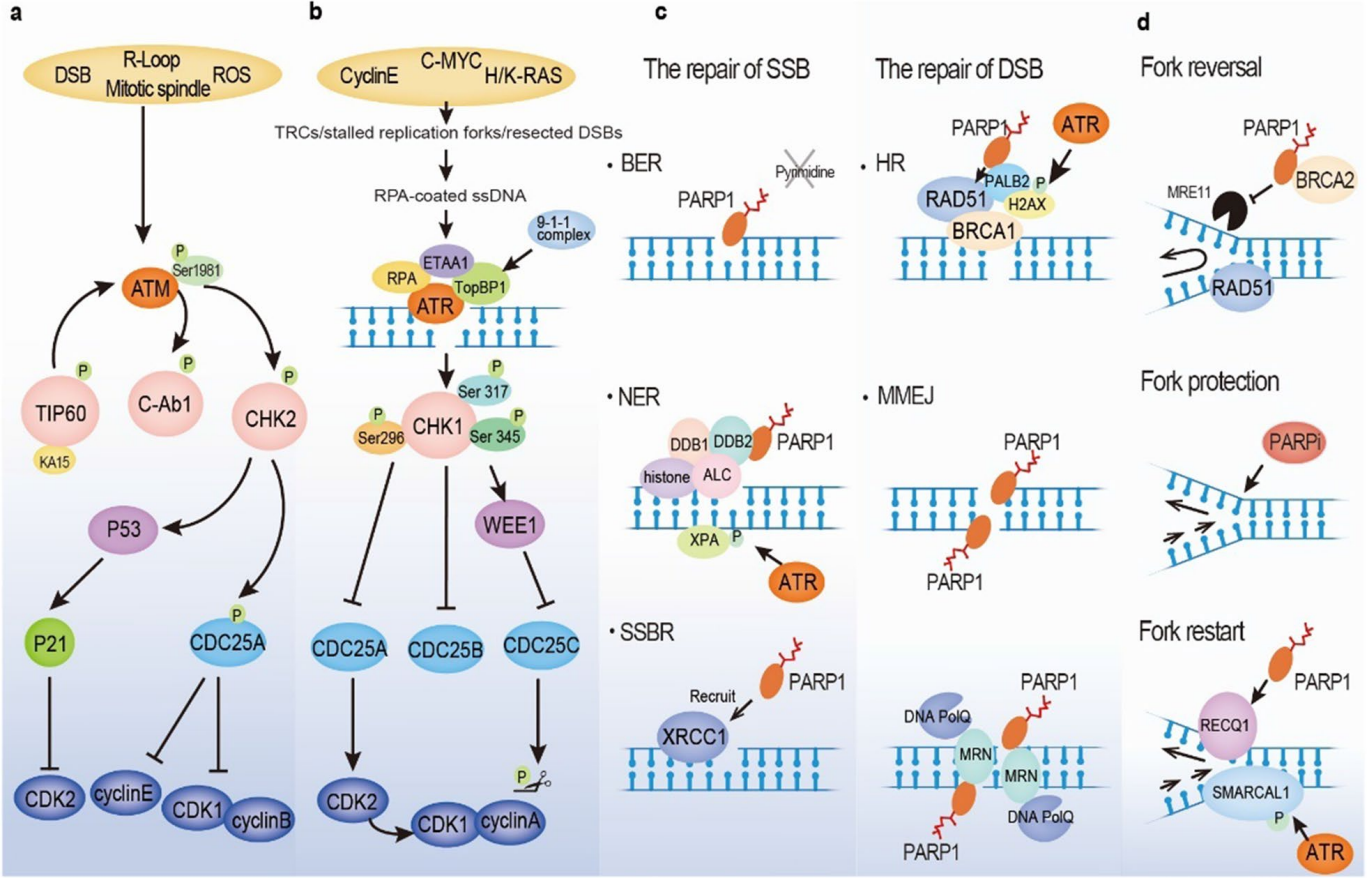
Multiple roles of two cell cycle checkpoint pathways (ATM/CHK2/TP53 and ATR/CHK1/WEE1) and PARP1 in the DNA damage response (DDR) and replication stress (RS) response. **a** ATM is stimulated by a few activators or via autophosphorylation through a feedback loop. ATM activates CHK2, which subsequently phosphorylates TP53 and CDC25A, leading to inhibition of CDK2/cyclin E and CDK1/cyclin B and impairment of G1/S phase transition. **b** Major trigger for ATR is stalled replication fork or resected DSB. The activation of ATR requires RPA-coated ssDNA, ETAA1, and TopBP1. Then, ATR mediates CHK1 phosphorylation, suppressing CDC25A and CDC25B and stimulating WEE1 activity. Alternatively, WEE1 inhibits CDC25C by phosphorylating CDC25C. The inactivation of the CDC family causes CDK2 and CDK1 incompetence, resulting in G2/M phase arrest. **c** PARP 1 senses single-strand DNA breaks and catalyzes other prominent members and itself through PARPylation, participating in BER, SSBR, and NER. Finally, poly(ADP-ribose) glycohydrolase (PARG) and PARylation itself release PARP1 from SSB sites. Similarly, PARP1 senses DSBs and recruits ATM, MRE11 and BRCA proteins to repair DNA strands via HR or facilitates MMEJ repair pathway activation in the absence of BRCA. **d** PARP1 plays key roles in fork reversal, fork protection and fork restart during the repair of stalled replication forks. *PARP1* Poly(ADP-ribose) polymerase 1; *DDR* DNA damage response; *RS* Replication stress; *BER* Base excision repair; *SSBR* Single-strand break repair; *NER* Nucleotide excision repair; *PARG* Poly (ADP-ribose) glycohydrolase; *SSBs* Single-strand breaks; *DSBs* DNA double-strand breaks; *ATM* Ataxia–telangiectasia mutant; *MRE11* Meiotic recombination 11 homolog 1; *BRCA* Breast cancer susceptibility gene; *HR* Homologous recombination; *MMEJ* Microhomology-mediated end joining

PARP1, a key member of the PARP family, is involved in many DDR pathways, such as the single-strand break repair (SSBR), HRR, and NHEJ pathways. Cell cycle checkpoints (ATM/CHK2/TP53 and ATR/CHK1/WEE1) not only sense G1/S and G2/M phase transitions but also play vital roles in phosphorylation as kinases during the DDR processes (Fig. 2). Although PARP2, the paralogue protein of PARP1, contributes merely 5%–10% PARylation activity in DNA damage response, it plays distinct and important roles in various DDR processes including BER, HR, a-NHEJ, and others [36–39]. As a member of DNA-dependent PARPs, PARP2 is composed of a specific WGR domain, a conserved C-terminal catalytic domain (CAT) and a compact N-terminal region (NTR) [40]. In particular, the NTR of human PARP2 is primarily responsible for DNA-binding affinity on DNA single-strand breaks and the other two domains are mainly required to recruit PARP2 for localization on DNA damage sites [40]. Notably, there is evidence suggesting that among three types of SSBs repaired by BER, namely nick, abasic site, and 1-nt gap, PARP2 shows a preference for binding to a nick over the others [37]. In addition, PARP2 also participates in orienting DSB repair choice to HR and alternative NHEJ pathways by limiting 53BP1 accumulation at DSB sites and facilitating DNA end resection, in which its PARylation activity is not a requirement [38]. Besides, PARP2 plays a key role in the repair process of flaps and gaps [38]. Interestingly, due to the WGR domain of PARP2 could span two DNA breaks and bridge them, PARP2 is also involved in nucleosome bridging and chromosomal modification [39]. Moreover, PARP2 is characteristic in being activated by 5’phosphorylated DNA breaks and mediating branched poly(ADP-ribose) chain synthesis in DNA repair [40, 41].

### The multidimensional roles played by PARP1 in the DDR

#### PARP1 regulates single-strand break repair

DNA base oxidation, alkylation damage, and abasic site generation commonly cause DNA SSBs in rapid succession, triggering excision repair mechanisms such as SSBR, BER, or NER [42, 43]. The PARP family members, particularly PARP1, play crucial and indispensable roles in the repair of DNA SSBs. PARP1, a ubiquitous nuclear enzyme, catalyzes the units of poly(ADP-ribose) (PAR) to specific amino acid residues in target proteins or to itself using *β*-NAD+ as a substrate via either linear or branched covalent ligation [42, 44]. Since PARP1 was identified as a pivotal participant in DDR in the 1980s, multiple researchers have studied its precise function in the BER pathway [13]. However, whether PARP1 plays a key role in this pathway has been debated [42]. PARP1 has been identified as a sensor and resolver of SSBs after incision of an abasic site (an AP site) during BER. However, purine base damage has recently been reported to be repaired via a PARP1-dependent BER pathway, whereas repair of pyrimidine base damage is independent of PARP1 [45]. Hence, the precise functions of PARP in BER warrant further investigation.

SSBs are rapidly detected by PARP1 within seconds of their creation. PARP1 mediates the ensuing PARylation of various other components of the SSBR, which include X-ray repair cross-complementing protein 1 (XRCC1), a core factor that acts as a scaffold for the SSBR complex and attracts them to SSB sites [46]. PARP inhibitors (olaparib and talazoparib) have been proven to induce synthetic lethality for the XRCC1-deficient phenotype in platinum-sensitive ovarian cancer. Furthermore, a large clinical trial revealed that high levels of XRCC1 and PARP1 are indicators of poor prognosis [47]. In addition to the scaffold protein XRCC1, unattached Okazaki fragments in the lagging strand have been implicated in PARP1-mediated SSBR [48]. UV damage is recognized by the DNA damage-binding protein 1 (DDB1)–DDB2 complex in the NER pathway. In addition, intrastrand cross-linked DNA, induced by cisplatin, which induces guanine–platinum–guanine or adenine compound formation, is repaired through the NER pathway [49, 50]. DDB2 binds and activates PARP1, which is followed by PARylation of histones and chromatin remodeling enzyme amplified in liver cancer 1 (ALC1, also known as CHD1L) activation. This in turn may facilitate DNA damage repair [51]. PARP1 is released at the site of an SSB via self-PARylation and poly(ADP-ribose) glycohydrolase (PARG) activity. A large number of ADP-ribosyl hydrolases, such as PARG, ARH1, MACROD1, MACROD2, and TARG1, have been identified to date [52, 53]. The aforementioned studies indicate the significance of PARP1 in DNA SSBR.

#### The function of PARP1 in DNA double-strand break repair

DSBs are commonly produced following exposure to DNA-damaging factors, such as ionizing radiation, and endogenous factors produced by the collapse of replication forks or programmed genome rearrangements [54]. However, DSBs formation can be propagated from SSBs via stalled replication forks [55]. PARP1 senses DSBs and recruits factors, such as ATM and meiotic recombination 11 homolog 1 (MRE11), which interacts with PARP-binding domains to repair the breaks [56, 57]. The role played by PARP1 in HRR seems contradictory to its other functions. On one hand, the BRCA protein is recruited to finish the repair of the first section of a DSB. RAD51 is recruited to the site via PARP1-dependent or DNA damage-mediated ubiquitylation [58]. However, the inhibition of PARP1 results in an elevated HRR rate as shown by an increase in sister chromatid exchange (SCE) and the number of RAD51 foci formed at DSB sites [59].

In addition to HR repair pathways for DSBs, there is currently a lot of interest in alternative nonhomologous end joining (a-NHEJ) repair pathway. A subset of a-NHEJ repair mechanism is mediated by microhomology sequences on either of DNA break ends, termed as microhomology-mediated end joining (MMEJ). Recently, it has been found that the majority of a-NHEJ relied on the DNA polymerase theta (POLθ; POLQ) function. The term polymerase theta mediated end-joining (TMEJ) was used [60]. The MMEJ pathway requires initial end resection near short DSB regions with microhomology (2–20 nucleotides), where PARP1 binds to DSB ends, recruits end resection factors, and facilitates MMEJ repair signaling. Without the initial end resection required for MMEJ, a Ku70-Ku80 heterodimer attaches to a DSB end, initiating classical-NHEJ (c-NHEJ) pathway activation [57, 61, 62]. Subsequently, PARP1, the MRN complex, and the peculiar DNA polymerase PolQ align complementary microhomologous sequences and facilitate DSB end bridging. The endonucleases (ERCC1/XPF, FEN1, and others not yet identified) remove the emerging nonhomologous 3’ tails, and the endonuclease FEN1 processes 5’ flaps. Then, PolQ fills the gaps in the DNA double helix caused by microhomology alignment and 3’ tail removal. Finally, the DNA ligase III/XRCC1 complex might be recruited by the MRN complex to DSBs to ligate the ends [61, 63, 64]. In particular, PARP1 interacts with DNA ligase III and the scaffold protein XRCC1 [63].

#### PARP1 modulates DNA replication forks

During the DDR process, replication fork reversal, protection, and restart are required to stabilize collapsed DNA replication forks [65, 66]. To gain more time to work on DNA break repairs, fork movement is first reversed. A four-way junction is formed at the site of the replication fork, which prevents the conversion of SSBs into DSBs [67]. Additionally, PARP1 and BRCA2 protect replication forks from MRE-11-induced degradation and preserve the stabilization of RAD51 foci [68–70]. Furthermore, the PARylation of RECQ1 via PARP1 mediates replication fork restart [69]. Hence, identifying the precise functions of PARP at DNA stalled replication forks is of great significance.

#### The role played by PARP1 in rDNA transcription and other processes

An RNA helicase, DDX21, that is PARylated by PARP1, is an essential factor for recombinant DNA (rDNA) transcription [71, 72]. Moreover, endogenous small nucleolar RNAs (snoRNAs) within the nucleolus activate PARP-1 enzyme activity, which is associated with the processing and modification of rRNA [71, 73]. PARP1 is also a chromatin remodeler that actives EP300, a histone acetyltransferase, resulting in the removal of nucleosomes by Brahma-related gene 1 (BRG1) and modulating the transcription of genes encoding the cell cycle and DNA repair [74]. Notably, in addition to its function in DNA repair, PARP1 plays a role in the induction of differentiation [75]. Overall, along with the multiple aforementioned roles played byPARP1, other mysterious biological functions of PARP1 require intensive exploration and further study.

### The ATM/CHK2/p53 pathway

#### ATM/CHK2/p53 pathway regulation of cell cycle checkpoints

Initially, the ataxia–telangiectasia mutated (ATM) gene was identified via the observation of ataxia–telangiectasia (A–T) patients, who present with gait disorder (ataxia), dilated capillaries (telangiectasia), and high sensitivity to cancer radiotherapy. Subsequent studies showed that when exposed to ionizing radiation (IR), DNA replication was not remarkably slowed in A–T cells, and defects were identified in the G1/S phase cell cycle checkpoint of these patients that were not found in healthy individuals [21]. As a member of the phosphatidylinositol 3-kinase-related protein kinase (PIKK) family, ATM exhibits a relatively conserved molecular structure that consists of HEAT repeats (~ 100–300 kDa *α*-solenoid polypeptide chains) in the N-terminus flanked by FATKIN domains, which contain the kinase domain (KIN) and its regulatory regions (FAT, PRD, and FATC domains) in the C-terminus and recognizes substrates. Recently, Stakyte et al. [76] revealed that HEAT repeats were composed of spiral and pincer domains, along with two zinc-binding motifs thought to be associated with the stability or binding of ATM. Although ATM autophosphorylation of Ser1981 in human A–T cells leads to its self-activation by mediating a dimer-to-monomer transition, this transition has also been induced by conformational changes in the presence of an activating ligand [76, 77]. Despite the presence of several distinct activators of the human ATM gene, such as DSBs, ROS, R-loops, and mitotic spindles, the activation canonical mode is mediated via a DSB-MRN (including MRE11, RAD50, and NBS1)-dependent pathway [78]. ATM is recruited to DSBs, stimulated by the DNA damage sensor MRN complex and subsequently phosphorylated by the target tyrosine kinase C-Ab1, which mediates the phosphorylation and activation of the TIP60/KAT5 acetyltransferase [76]. The TIP60/KAT5 acetyltransferase participates in the posttranslational modification of ATM by promoting its autophosphorylation at Ser1981, apparently forming a positive feedback loop promoting the regulation of ATM activity [76, 78]. The ATM/CHK2/p53 pathway primarily controls the G1/S checkpoint and is partially involved in G2/M checkpoint regulation by inhibiting CDK activity. CHK2 is phosphorylated by activated ATM and in return, CHK2 stimulates p53, which subsequently activates the CDK2 inhibitor p21 thereby causing G1 phase arrest and impairing cell cycle entry into the S phase [15]. Additionally, the CHK2 kinase mediates the activation of CDC25A. CDC25A phosphatase removes a phosphate moiety from the CDK2/cyclin E complex, activating the complex. In contrast, phosphorylation of the CDK2/cyclin E complex deactivates it and causes cell cycle arrest. Moreover, as a member of the CDC phosphatase family, CDC25A dephosphorylates the CDK1/cyclin B complex and has also been proven to be a substrate of CHK2, resulting in halting of the cell cycle before mitosis [79]. Overall, ATM, CHK2, and p53 are paramount for monitoring DNA damages and slowing the progression of the cell cycle, especially in the G1 phase.

#### The ATM/CHK2/p53 signaling pathway participates in DNA damage repair

Apart from monitoring cell cycle checkpoints, ATM also participates in the repair of DSBs and SSBs. ATM is involved in topoisomerase adducts formation as well [78]. ATM performs a dual role in the DSB repair pathway by acting as a kinase, both HR and NHEJ repair pathways triggered on the basis of DNA end resection [21]. For DSBs with obstructed ends or heteromatism, ATM phosphorylates the histone variant H2AX, known as γH2AX, at Ser139 in the C-terminal tail. ATM simultaneously recruits CtBP-interacting protein (CtIP) to the damage site and mediates the phosphorylation of CtIP at Thr859, which together launch DNA end resection [15, 21, 80]. In addition, exonuclease 1 (EXO1) and DNA2, which mediate short- and long-range resection, have been confirmed targets of ATM [81].

### The ATR/CHK1/WEE1 pathway

#### ATR/CHK1/WEE1 pathway regulation of cell cycle checkpoints

Cancer cells driven by oncogenes are hyperdependent upon the ATR/CHK1/WEE1 signaling pathway, which enables them to attenuate RS. A newly described model has indicated that RS is a novel hallmark of cancer progression because it is an outcome of oncogene mutation which induces continuous proliferation, genome instability, and inhibited apoptosis [82]. Intriguingly, Panagiotis et al. drew an analogy between the interwoven pathways of oncogene-induced RS and scattered jigsaw puzzle pieces, with the most significant piece being transcription deregulation or TRCs involving cyclin E, c-MYC, or H/K-RAS upregulation. RS was highlighted as a driving force of oncogenesis, especially in precancerous lesions [4, 83–85]. As a primer–template junction composed of ssDNA, RPA, and, as recently discovered, double-stranded DNA (dsDNA), the ATR/CHK1/WEE1 pathway may trigger an RS response, recruiting related proteins to stabilize and restart the replication fork, in which its major player, ATR, helps to maintain the genome stability [86–90].

Given that the majority of cancers harbor the pervasive TP53 mutation, which results in dysfunction of the G1 checkpoint, cancer cell is thought to rely heavily on S and G2/M cell cycle checkpoints, which gives the DNA damages enough time to repair via RS response. This process is proposed to be regulated mainly by the ATR/CHK1/WEE1 pathway [91]. ATR is a crucial member of the PIKK family and it is activated by RPA-coated SS DNA. Its most important role is in RS and lesions in replicating DNA [91–93]. Mice completely lacking ATR function have been reported to present early embryonic lethality [94]. On one hand, RPA coats ssDNA, preventing it from degradation and from interacting with ETAA1, an ATR activator that localizes to ssDNA [95]. Additionally, the ATR–interacting protein (ATRIP) complex is required for ATR activity [96]. Furthermore, another ATR activator, DNA topoisomerase 2-binding protein 1 (TopBP1), is simultaneously recruited by the 9-1-1 complex (RAD9-RAD1-hust) to activate ATR [95, 96]. Once activated, ATR phosphorylates multiple downstream proteins, the most well-known of which is CHK1, which is phosphorylated at Ser317 and Ser345 [95]. Notably, the autophosphorylation of CHK1 at Ser317 is an ATR-independent modification that prevents replication disruption after treatment with ATR inhibitors (ATRi) treatment [97]. As mentioned above, compared to normal cells, cancer cells are more dependent on S and G2/M checkpoints due to the loss of G1 checkpoint control [98]. In the CDC25 phosphatase family, both CDC25A and CDC25B are inactivated by CHK1 phosphorylation activity. CDC25A dephosphorylates CDK2, while CDC25B removes a phosphate moiety from CDK1, leading to S and G2/M arrest, respectively [99]. Furthermore, activated CHK1 activates WEE1 via phosphorylation, leading to inactivated CDC25C and precluding G2/M phase progression through CDK1 [100]. In contrast, the administration of small-molecule inhibitors, such as ATRi, CHK1i, or WEE1i, to cancer cells that regularly experience replication stress can lead to shortened S and G2/M arrest duration and chromosomal aberration, replication catastrophe, and even cell death.

#### The ATR/CHK1 axis participates in DNA damage repair

In addition to the regulation of cell cycle checkpoints, ATR/CHK1 participates in DNA damage repair through NER, HRR, and replication fork stabilization. XPA, a key NER factor, is phosphorylated by ATR, resulting in XPA localized recruitment to DNA damage sites [101, 102]. Moreover, ATR is associated with chromatin and HRR-related proteins, including BRCA1, PALB2, RAD51, and H2AX (via ATR itself or indirectly via CHK1) [103]. Intriguingly, RAD51 is also directly phosphorylated by CHK1, which is a requirement for DNA filament formation in the HRR signaling pathway [99, 104, 105]. ATR is involved in correcting chromosome instability in mitosis by accelerating chromosomal segregation, which is completely different from its role in RS [106]. Regarding replication fork stability, ATR activates the helicase SMARCAL1 by phosphorylating Ser652, which restarts a stalled replication fork [107]. To sum up, ATR and CHK1 are core members of the DDR pathway and are involved in phosphorylation and various DNA repair signaling pathways.

## The mechanisms underlying PARP inhibitor resistance: three and counting

**Fig. 3:**
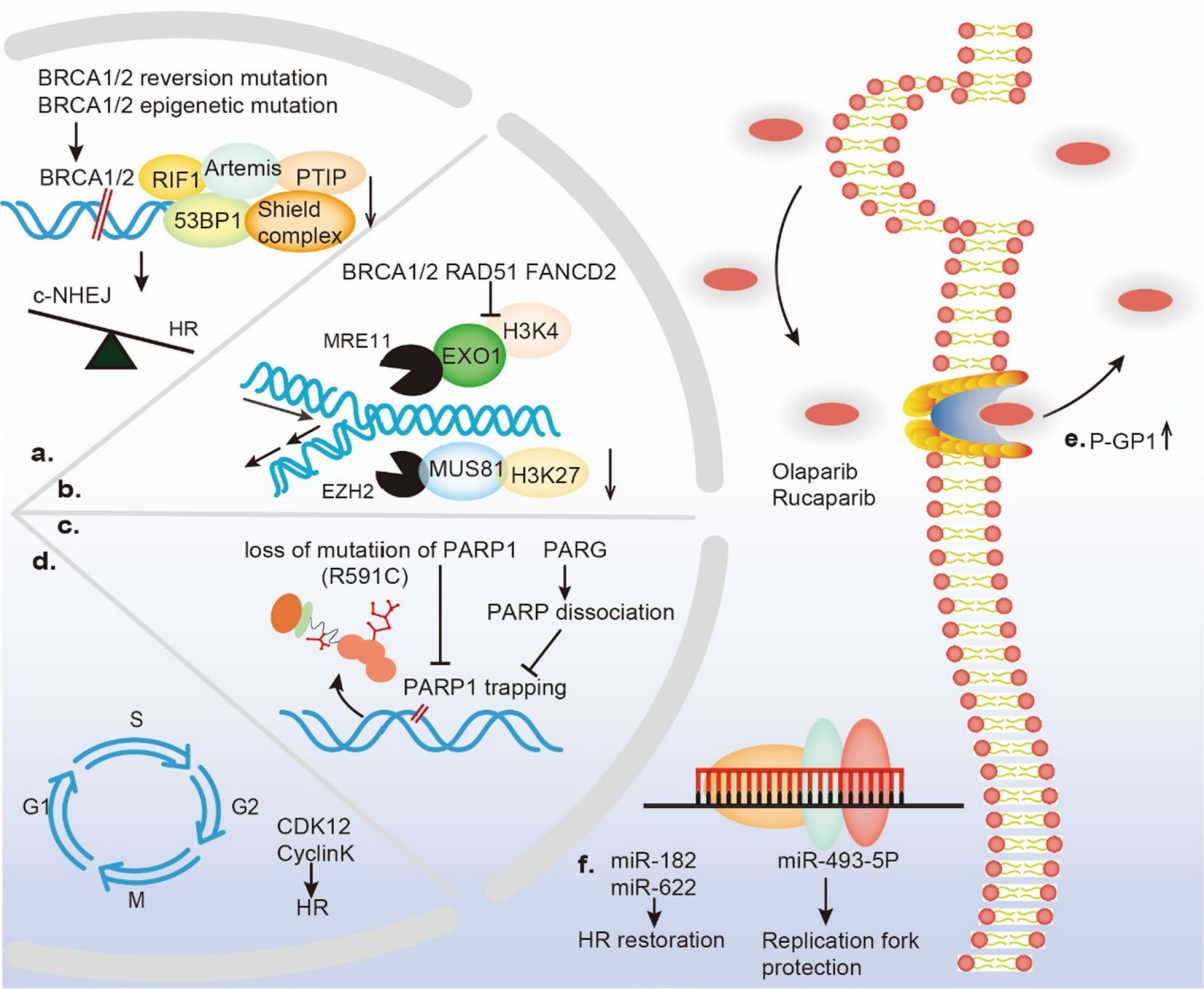
Potential mechanisms of PARP inhibitor resistance. In a clinical setting, the mechanisms of resistance to PARP inhibitors (PARPi) present heterogeneity and mainly consist of **a** restoration of homologous recombination (HR) ability; **b** protection of replication fork stability; **c** reduction in PARP1 trapping; **d** mutations in cell cycle components; **e** efflux of the PGP-mediated drug pump; **f** interference in the microRNA (miRNA) environment

In general, the mainstream mechanisms of PARP inhibitor resistance can be categorized as HR-dependent or HR-independent. The restoration of HR competence has been proven to be relevant to BRCA1/2 secondary or reversion mutations, loss-of-53BP1, Shieldin complex mutations, and genetic alterations associated with DSBs repair. In PARP inhibitor resistance mechanisms that function independently of HR, the protection of stalled replication forks is favored due to defects in the nucleases MRE11 and MUS81. In addition, a decrease in PARP1-trapping, augmentation of drug efflux, dysregulation of cell cycle-associated molecular mechanisms, and expression of microRNAs (miRNAs) mediate resistance to PARP inhibitors independently of the HRR pathway (Fig. 3).

### Restoration of HR ability

In a clinical setting, the most ubiquitous mechanism of acquired PARP inhibitor resistance is DDR rewiring through BRCA secondary reversion mutations, decreasing the expression level of 53BP1 and other components, such as the Shieldin complex, RIF1, REV7, PTIP, and Artemis [108–112]. In 2008, two of the three types of human recurrent ovarian tumors displaying mutations restoring an open reading frame (ORF) function and loss of the mutant BRCA2 allele were reported, and the outcomes were a corrected frameshift of BRCA reading frame [113]. Deep parallel DNA sequencing during, before, and after olaparib treatment of primary stage III high-grade serous ovarian cancer (HGSOC) tumors performed by Barber et al. revealed that secondary 4-bp and 12-bp deletions during lymph node metastasis restored the full-length BRCA2 protein structure and function [114]. Additionally, BRCA epigenetic mutation (loss of BRCA1 promoter methylation) is not a genetic alteration but leads to the functional recovery of BRCA1 protein expression [115]. Taken together, the functional restoration of BRCA via genetic or epigenetic mutations is a significant factor for regaining HR capacity.

The status of 5’-end DNA resection is a determining factor in the mechanism by which DSBs are repaired. The resection of the DNA 5’-end produces 3’ overhangs that are required for HRR. Without 5’-end DNA resection, DNA is unprotected, triggering c-NHEJ repair pathway. The guardian at the 5’-end has been identified as the 53BP1/RIF/Shieldin complex (Rev7/Shld1/Shld2/Shld3), which functions in a CST (Ctc1, Stn1, Ten1)/polα-dependent manner [116–118]. A RING domain mutation and exon 11 in BRCA1 have often been reported in familial ovarian cancer patients whose HRR competence was restored with 53BP1 deficiency, leading to PARP inhibitor resistance [119, 120]. Furthermore, once transduced by single guide RNAs (sgRNAs) targeting Shld1 and Shld2, breast cancer cells with BRCA1 deficiency showed a diminished response to PARPi, similar to the results of in vivo experiments [118]. Shieldin subunit gene disturbance is a hallmark of ovarian cancer, and an explanation of this outcome requires further exploration.

In addition to the HR and c-NHEJ repair pathways for DSBs, interest in the MMEJ repair pathway has been recently observed. An increasing number of studies have shown that the relations between the MMEJ and HR pathway components are competitive. When either the HR or MMEJ repair pathway was triggered, MMEJ accounted for 10%–20% of the repair realized through HRR in normal mammalian cells [64]. However, HRD cancer cells have been found to be highly dependent on MMEJ to repair DSBs. The N-terminal helix domain of the MMEJ-specific DNA polymerase PolQ shows ATPase activity, which may facilitate RAD51 binding and prevent toxic RAD51-ssDNA filament assembly. Moreover, the unstructured central region of PolQ includes a RAD51 interaction motif that inhibits toxic RAD51 accumulation along resected ssDNA tracts to promote MMEJ repair and prevent HRR. In addition, the ATPase activity of PolQ may lead to the removal of RPA from ssDNA to allow MMEJ repair [63]. Indeed, in a backup DSB repair pathway (MMEJ), PolQ was found to be overexpressed in HRD and PARP-inhibitor-resistant tumor cells. A newly identified PolQ inhibitor Novobiocin resensitized these cancer cells to PARPi in vitro and in vivo by increasing the excessive DSB end resection and nonfunctional RAD51 foci-loading rates in HRD and PARP-inhibitor-resistant tumor cells [121]. The mechanisms of PARP inhibitor resistance in these tumors involve a decrease in 53BP1 or the Shieldin complex and the stabilization of replication forks [121]. Overall, PolQ is an exploitable drug target to overcome PARP inhibitor resistance.

### Protection of fork stability

In addition to the restoration of HR capacity, protection of replication fork stability, mainly by crippling nuclease activity, is the main mechanism of acquired PARP inhibitor resistance [122, 123]. In RS, stalled replication forks tend to create a four-way structure composed of two template strands and two nascent DNA strands. Several DNA translocases, such as SMARCAL1, ZRANB3, and HLTF, catalyze fork reversal by acting as replication fork remodelers that activate the nuclease MRE11 to degrade the nascent DNA, while another nuclease, EXO1, potentiates fork degradation [122, 124]. However, PARP1, BRCA1/2, RAD51, and the Fanconi anemia (FA) gene FANCD2 protect nascent DNA from the nucleolytic activity of MRE11. Notably, in BRCA1/2-deficient breast cancer cells, the loss of fork remodelers protected replication forks from collapse, rescued genome stability, and induced resistance to PARPi [122, 123, 125]. The nucleolytic activity of MRE11 has been associated with H3K4 (Lys4 on histone 3) methylation [126]. Moreover, enhancer of zeste homolog 2 (EZH2) has been confirmed to be located at stalled replication forks with Lys27 on histone 3 found to be trimethylated (H3K27me3 mark), was recruited the endonuclease MUS81, contributing to stalled fork degradation both in murine and human models with BRCA2-null tumors. Downregulation of the EZH2/MUS81 pathway protects the stability of reversed forks and predicts PARP inhibitor resistance in tumors with deleterious BRCA-2 mutations independent of the nuclease MRE11-associated pathway [123]. In brief, diminished nuclease MRE11/EXO1 or endonuclease EZH2/MUS81 axis activity protects the stability of replication forks against RS to some extent, resulting in PARP inhibitor resistance.

### Reduction in PARP1 trapping

In addition to inhibiting the enzymatic activity of PARPs, PARPi kills cancer cells through a mechanism termed as “PARP trapping,” in which a PARP inhibitor physically traps PARP protein on damaged DNA [127–129]. These PARP–DNA complexes may form toxic steric barriers to replication fork progression, eventually leading to abnormal chromatin formation during mitosis and cell death in HRD tumors [129, 130]. Through genome-wide and focused screening of PARP1 using CRISPR‒Cas9 technology, research has shown that the impaired cytotoxicity of PARPi in BRCA1-mutant triple-negative breast cancer (TNBC) and ovarian cancer cells was related to the loss or mutation of the PARP1 protein by abolishing PARP1 trapping. A study with a patient presenting de novo resistance to olaparib because of the PARP1 mutation R591C, which led to the rapid dissociation of PARP1 from damaged DNA sites, confirmed the above-mentioned genetic finding [131]. Furthermore, although no mutations were found within or outside the DNA-binding domain of PARP1, other factors exerting a trapping effect on PARP1 confer resistance to PARPi. Remarkably, PARP1 can be released through self-PARylation, in which poly(ADP-ribose) glycohydrolase (PARG) performs an inverse function [52, 53]. PARG deletion or inhibition was confirmed to hinder PARP1 function in chromatin binding due to increased PARylation and countered PARP inhibitor cytotoxicity by accelerating the dissociation of PARP1 from ssDNA [132]. Notably, due to the loss of PARG in vivo, pre-resistance to PARPi has also been found in serous ovarian cancer biopsied samples, with parallel findings reported in vitro [133]. Taken together, these studies suggest that PARP1-DNA trapping may be reduced by decreasing PARP1 binding or promoting PARP1 dissociation from chromatin, which suggests a mechanism that differs from that involving HR restoration or protection from replication forks.

### Efflux via a PGP-mediated drug pump

P-glycoprotein (PGP, encoded by the ABCB1 gene), also known as multidrug resistance protein 1 (MDR1), physiologically safeguards cells against intrinsic and extrinsic toxic agents, such as anticancer drugs [134, 135]. The expression of MDR1 was found to be low in ovarian carcinomas when BRCA1-deficient mammary tumors were diagnosed initially, whereas the upregulation of this pump protein always occured in parallel with acquired resistance to chemotherapy, including PARPi [132, 136]. Accordingly, the coadministration of the P-glycoprotein inhibitor tariquidar along with olaparib could counteract acquired PARP inhibitor resistance in TNBC models [132].

An increase in the copy number of ABCB1 was a result of the amplification of specific regions on chromosome 7q21 in ovarian cancer, which was a consistent finding in several studies [137–139]. A whole-genome sequencing (WGS) study reported that in 92 patients with primary and matched resistant HGSOC, the level of ABCB1 gene upregulation was associated with promoter fusion and translocation at the 5’-region of the gene [136, 140]. Additionally, certain PARP inhibitors, including olaparib and rucaparib, are P-glycoprotein substrates that are actively secreted from paclitaxel-resistant cells. However, other drugs such as veliparib and AZD2461, are not suitable substrates for P-glycoprotein. For example, AZD2461 was not readily transported by PGP [137]. Given that several clinical trials concerning MDR1 inhibitors have produced disappointing results (e.g., NCT00069160 and NCT00001944), this resistance mechanism should be studied further using fourth-generation agents derived from natural sources that show high efficiency and nontoxicity [141].

### Mutations of cell cycle components

Molecules of the cell cycle, such as CDK12 and cyclin K, inhibit cell sensitivity to PARPi by influencing the HRR pathway. In a genome-wide screening study on PARP1/2 inhibitors, the silencing of CDK12 using a short hairpin RNA (shRNA) construct conferred a modest but significant increase in sensitivity to olaparib coupled with a reduction in 53BP1 foci formation at a scale equivalent to that achieved via BRCA1 attenuation, which resulted in HR deficiency. Furthermore, a similar effect has also been observed after CCNK silencing, in which cyclin K is a regulator of its partner CDK12 [142]. In contrast, based on the overexpression of CDK12 and CCNK, these molecules may be considered determinants of PARP inhibitor resistance and are of paramount importance for identifying clinical alternatives for specific therapeutic interventions that can be used with recurrent ovarian cancer patients. The aforementioned preliminary investigation demonstrated that somatic mutations of the CDK12 domain abrogated the kinase activity of CDK12 and inhibited reduction in a subset of HR-associated molecules, including BRCA1 (as illustrated above), ATR, FANCI, FANCD2, and RAD51 foci, inducing sensitivity to PARPi [143]. Collectively, the overexpression of cell cycle components, particularly CDK12 or cyclin K, indirectly diminished the sensitivity to PARPi to some extent in tumor cells.

### Interference by microRNAs (miRNAs)

Several studies have illustrated that miRNA expression is associated with the repair of DSBs or stalled replication forks, which are involved in resistance to PARPi. The miR-182 plays a prominent role in impeding the expression of BRCA1, which is of significant clinical relevance. This is because antagonizing miR-182 confers cell resistance to PARPi due to enhanced HR ability via the upregulation protein expression of BRCA1 [144]. Strikingly, in ovarian cancer with loss of BRCA1, miR-622 impaired the NHEJ pathway but rescued HR-mediated DSB repair via downregulation of the ku70/ku80 complex during the S phase by inducing resistance to PARPi [145]. The role played by miR-622 in determining the initiation of NHEJ or HR is a recently identified mechanism in PARP inhibitor resistance, and therefore, miR-622 is a promising biomarker for evaluating the efficacy of PARPi in BRCA1-inactivated epithelial ovarian carcinomas (EOCs). The overexpression of miR-493-5p, a miRNA that acts as a mediator of PARP inhibitor (including olaparib and rucaparib) resistance, is relevant to the preservation of replication fork stability that results from the diminished nuclease activity of MRE11, CHD4, and EXO1 evident exclusively in BRCA2-mutated ovarian carcinomas [146]. As mentioned above, similar to those of cell cycle molecules, expression alterations of specific miRNAs influence the ability of PARPi via HR-dependent or replication forks-protected pathways.

## An array of cancer cell lines that harbor PARP inhibitor resistance

**Fig. 4:**
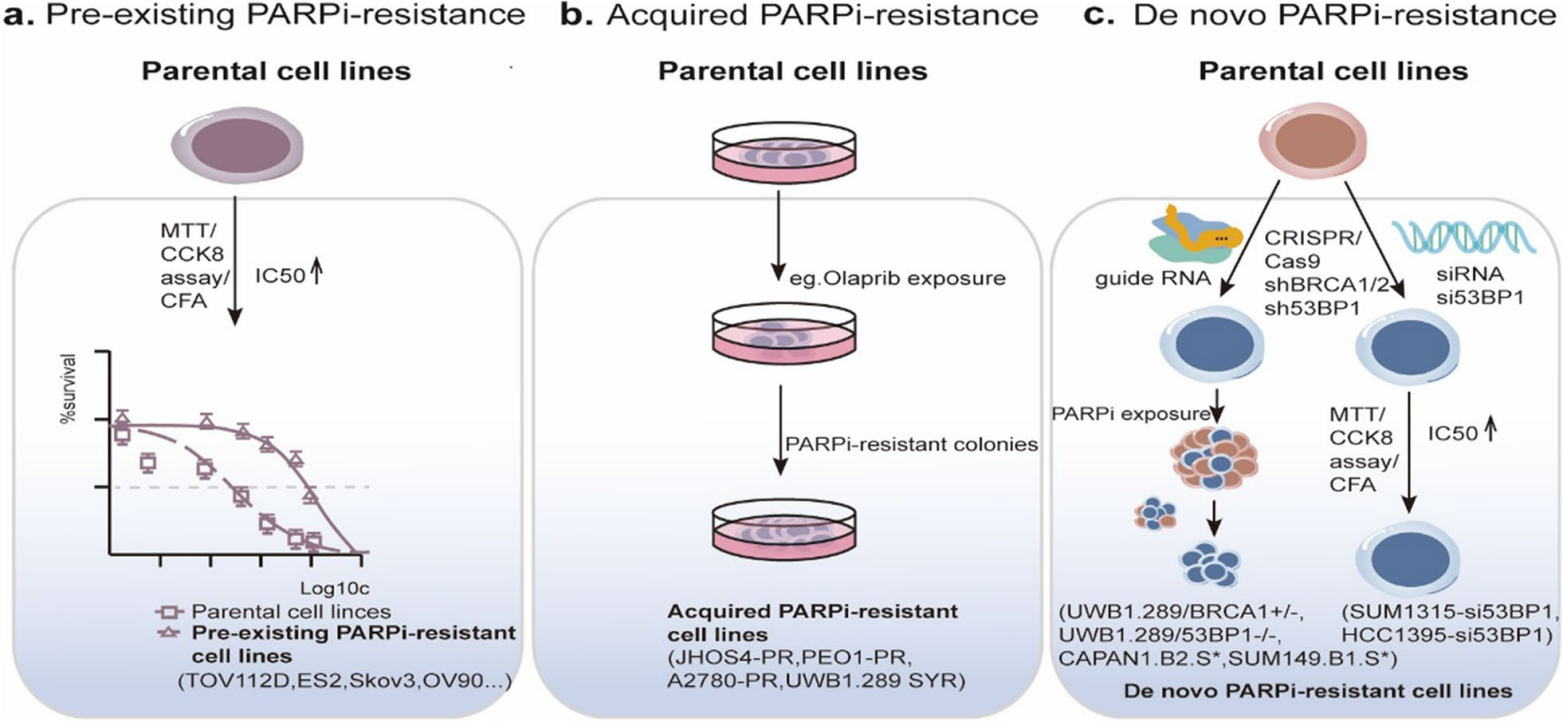
Approaches for building PARP-inhibitor-resistant cancer cell lines. In preclinical trials, the construction of PARP inhibitor-resistant cancer cell lines was performed primarily by exploiting the following three mechanisms: **a** preexisting intrinsic PARP inhibitor resistance; **b** acquired PARP inhibitor resistance under extended and constant PARP inhibitor exposure; **c** de novo PARP inhibitor resistance via knockout or knockdown of key genes related to PARP inhibitor resistance. *PARPi-resistance* PARP inhibitor resistance; *MTT* 3-(4,5-dimethylthiazol-2-yl)-2,5-diphenyltetrazolium bromide; *CCK-8* Cell counting Kit-8; *CFA* Colony formation assay

Intriguingly, three different types of PARP inhibitor resistance have been established, namely preexisting PARP inhibitor resistance, acquired PARP inhibitor resistance, and de novo PARP inhibitor resistance. Nearly one-half of ovarian cancer patients are HR-proficient and do not respond well to olaparib, hinting PARP inhibitor resistance before the administration of olaparib treatment [147]. Tumor cells often acquire resistance during prolonged exposure to PARPi. The suppression or knockdown of specific genes is the primary cause of de novo PARP inhibitor resistance (Fig. 4).

Therefore, to explore and overcome various PARP inhibitor resistance mechanisms, researchers have constructed an array of drug-resistant tumor cell models based on the three PARP inhibitor resistance mechanisms. Based on the concept of a preexisting mechanism, several tumor cell lines were found to be intrinsically resistant to PARP inhibitor treatment involving BRCA-wild-type TOV112D, ES2, SKOV-3, and OV-90 cells, which was identified via elevated half-maximal inhibitory values (IC50) of olaparib [105, 148]. Due to an increase in the application of PARPi in clinical regimens, acquired resistance can be expected to be common. Similarly, after prolonged exposure to a PARP inhibitor-exposed environment, several cancer cell lines, such as the JHOS4-PR, PEO1-PR, and UWB1.289 SYR cell lines, evolved acquired resistance compared to their parental counterparts. The requisite time to acquire PARPi resistance ranged from several months to nearly 1.5 years [149]. Given that the restoration of BRCA, loss of 53BP1, and even the amplification of XPC conferred resistance to PARPi in a diverse set of cancer cells, CRISPR‒CasCas9, a genome-editing technique has been exploited to establish de novo PARP inhibitor resistance [105, 148]. Notably, UWB1.289/BRCA1 ± PARP-inhibitor-resistant ovarian cells, the CAPAN1.B2. S* pancreatic ductal adenocarcinoma cancer (PDAC) cell line (harboring BRCA2 secondary mutations, c.[6174delT;6182del5]), and the SUM149.B1.S* mammary tumor cell line (with secondary BRCA1 mutations, c.[2288delT; 2293del80]) were insensitive to PARPi after CRISPR mutagenesis was used to reconstitute BRCA function [150, 151]. In contrast, another approach for driving de novo resistance was the knockdown of 53BP1 in BRCA1-mutant ovarian cancer cells, namely the UWB1.289 cell derivatives UWB1.289/53BP1−/− cells. SUM1315 breast cancer cells and HCC1395 breast cancer cells, which were generated via the use of short interfering RNA against 53BP1 (si53BP1) [150, 152]. Collectively, the construction of PARP-inhibitor-resistant cancer cell models in vitro was the first and critical step for preclinical studies and can be expanded to the construction of PARP-inhibitor-resistant models in vivo.

Intriguingly, a certain level of heterogeneity characterized different PARP-inhibitor-resistant cancer cell lines, and multiple distinct drug resistance mechanisms may be involved in the same model cell line. In de novo PARP-inhibitor-resistant tumor cells, the restoration of HR is one mechanism, as has been demonstrated after secondary mutation of the BRCA protein in UWB1.289, CAPAN1, and SUM149 parental cells or the knockdown of 53BP1 in UWB1.289 and COV362 cells [105, 149, 150]. However, in other acquired PARP-inhibitor-resistant UWB1.289 or PEO1 cell lines, the reversion of BRCA mutation was not detected, and only a few cell models showed the loss of 53BP1 or upregulation of the MDR protein [149, 150]. Epigenetic alterations at DNA damage checkpoints are common in a broad spectrum of cancer cells and involve epigenetic regulators, such as EZH2, ANKRD30, RARA, and HOMEZ. Taken together, these studies have identified genome instability and chromosome rearrangement in certain PARP-inhibitor-resistant cells, which indicates that other drug resistance mechanisms have yet to be elucidated [150].

## Combined cell cycle checkpoint blockade with PARP inhibitors to overcome PARP inhibitor resistance: preclinical data

Overall, a combinational strategy in which cell cycle checkpoint inhibitors and PARPi are simultaneously delivered to a series of cancer cell models has proven to be considerably promising over the past few decades. Owing to the high mutation rate of the ATM/CHK2/p53 pathway genes, the regimen tends to focus on targeting PARP and ATM/CHK2/p53 intrinsic alterations (Table 1). Besides, applications of ATR/CHK1/WEE1 small-molecule inhibitors plus PARP inhibitors have been suggested, especially for cancer cells resistant to PARPi (Table 2).

**Table 1:** Preclinical trials of ATM-CHK2-p53 alterations and PARPi in cancer cell lines

Histological type	Cell line	Mutations	Cell cycle inhibitors/alterations + PARPi	Synergism	Effect of combo on ATM/CHK2/p53	Effect of combo on DSBs	Effect of combo on HR	Effect of combo on replication forks	Cell cycle alterations	Effect of combo on apoptosis	References
Prostate	22Rv1 CL1-8	ATM-MUT	ATM-MUT + PARPi + ATRi (ATM-MUT + Rucaparib + VE-822)	Yes	–	↑	↓	–	No	–	[129]
	22Rv1 LNCaP DU-145	ATM-WT	ATMi + PARPi + ATRi (Ku-60019 + Rucaparib + VE-822)	Yes	↓(pCHK2 T68 and pCHK1 S345)	↑	↓	–	No	–	
Pancreas	AKC	ATM-loss	ATM-loss + PARPi + ATRi + DNA-Pki (ATM-loss + Olaparib + VE-822 + CC-115)	Yes	–	↑	–	Fork asymmetry;↓	–	↑	[132]
	AKC995	ATM-loss	ATM-loss + PARPi ± IR/Gimcitabine (ATM-loss + olaparib/niraparib + IR/ Gimcitabine); ATM-loss + ATRi (ATM-loss + VE-822)	Yes	–	↑	↓	↓	–	↑	[130]
	AKC5615 AKC5980 AKC5982	ATM-loss	ATM-loss + PARPi ± IR/Gimcitabine (ATM-loss + olaparib/niraparib ± IR/ Gimcitabine); ATM-loss + ATRi (ATM-loss + VE-822)	Yes	– (p-CHK1↑ p-KAP1↑)	–	–	–	–	–	[130]
	ATM^+/+^ MIA PaCa-2	ATM^+/+^	ATMi + PARPi + ATRi + DNA-PKi (AZD0156/KU60019 + Olaparib + VE-822 + CC-115)	Yes	–	↑	–	–	–	–	[132]
	ATM^+/Δ^ MIA PaCa-2	ATM^+/Δ^	ATM + /Δ + PARPi + ATRi + DNA-Pki (ATM + /Δ + Olaparib + VE-822 + CC-115)	Yes	–	↑	–	–	–	–	[132]
	ATM^Δ/Δ^ MIA PaCa-2	ATM^Δ/Δ^	ATMΔ/Δ + PARPi + ATRi + DNA-PKi (ATMΔ/Δ + Olaparib + VE-822 + CC-115)	Yes	–	–	–	–	–	–	[132]
Lymph	PGA-shATM	ATM-loss	ATM-loss + PARPi (ATM-loss + Olaparib)	Yes	–	–	–	–	–	–	[153]
	Granta-519	ATM-MUT	ATM-MUT + PARPi (ATM-MUT + Olaparib)	Yes	–	–	–	–	–	–	[153]
Head and neck	FaDu	ATM-KO	ATM-KO + PARPi + ATRi (ATM-loss + Olaparib + AZD6738)	Yes	↓	↓(γH2AX↓;micronuclei↑)	–	–	G2/M↓	↑	[154]
Nasopharyngeal	HONE1	ATM-WT	ATMi + PARPi (KU-60019 + Olaparib)	Yes	↑	↑	↓	–	Cell cycle arrest; G2/M↑	–	[155]
			ATMi + PARPi (KU-60019 + Veliparib)	Yes	↑	↑	–	–	G2/M↑	–	[155]
Lung	A549	ATM-KO; TP53-WT	ATM-KO + PARPi + ATRi (ATM-KO + Olaparib + AZD6738);	Yes	–	–	–	–	–	↑	[154]
			ATM-KO + PARPi (ATM-KO + Olaparib/Talazoparib)	Yes	↑	↑	–	–	G2↑	↑	[156]
	NCI-H2	ATM-MUT	ATM-MUT + PARPi + ATRi (ATM-MUT + Olaparib + AZD6738	Yes				–	–	↑	[154]
Kidney (Embryo)	HEK293	ATM-WT	ATMi + PARPi (AZD0156 + Olaparib)	Yes	–	↑	↑	–	–	↑	[157]
Cervix	Hela	p53-deficiency	CHK2i + PARPi (CCT24153 + Olaparib)	Yes	↓	–	–	–	–	↑	[133]
			CHK2i + PARPi (CCT241533 + Rucaparib)	Yes	–	–	–	–	–	–	[133]
Colon	HT-29	p53-deficiency	CHK2i + PARPi (CCT241533 + Rucaparib)	Yes	–	–	–	–	–	–	[133]
Breast	MDA-MB468	Mtp53 273H; Mtp53248W (Gain of function; GOF)	TP53-MUT(GOF) + PARPi + temozolomide (TP53-MUT(GOF) + Talazoparib + temozolomide)	Ye	–		↓	–	–	–	[137]
	MCF-7	TP53-WT	TP53-WT + PARPi + Irradiation (TP53-WT + Veliparib + Irradiation)	Yes	–	–	–	–	–	–	[139]
	MCF-7	Mtp53 273H(GOF)	TP53-MUT(GOF) + PARPi + Irradiation (TP53-MUT(GOF) + Veliparib + Irradiation)	Yes	–	–	–	–	–	–	[139]
Bladder	UMUC3	TP53-MUT(F113C)	TP53-MUT + PARPi (TP53-MUT + Olaparib)	Yes	–	↑	No	–	–	–	[138]
	5637	TP53-MUT(R280T)	TP53-MUT + PARPi (TP53-MUT + Olaparib)	Yes	–		No	–	–	–	[138]
	639v	TP53-MUT(R248Q)	TP53-MUT + PARPi (TP53-MUT + Olaparib)	Yes	–	↑	No	–	G1↑		[138]
Colorectum	SKCO1	TP53-WT	TP53-WT + PARPi (TP53-WT + Talaparib)	Yes	↑		↓				[139]
	LS513	TP53-WT	TP53-WT + PARPi (TP53-WT + Talaparib)	Yes	↑					–	[139]
	HCT116	TP53-WT	TP53-WT + PARPi + RMRP-sg-1 (TP53-WT + Olaparib + RMRP-sg-1)	Yes	–	–	–	–	–		[138]
	LOVO	TP53-WT	TP53-WT + PARPi + RMRP-sg-1 (TP53-WT + Olaparib + RMRP-sg-1)	Yes	–	–	–	–	–	–	[138]
Cerebrum	U87	TP53-WT	TP53-WT + PARPi + Irradiation (TP53-WT + Veliparib + Irradiation)	Yes	–	–	↓	–	–	–	[139]
	SF767	TP53-WT	TP53-WT + PARPi + Irradiation (TP53-WT + Veliparib + Irradiation)	Yes	–	–	–	–	–	–	[139]

*PARPi* PARP inhibitors; *Combo* Combination; *ATM-MUT* ATM mutant; *ATM-WT* ATM wild type; *ATM-KO* ATM knockout; *TP53-WT* TP53 wild type; *GOF* Gain of function; *ATRi* ATR inhibitors; *ATMi* ATM inhibitors; *DNA-PKi* DNA-PK inhibitors; *IR* Irradiation; *p-CHK2* Phosphorylated CHK2; *p-CHK1* Phosphorylated CHK1; *p-KAP1* Phosphorylated KAP1; *ATM*^+*/*+^Only the internal fragment was amplified; *ATM*^+*/Δ*^ Heterozygous ATM deletion; *ATM*^*Δ/Δ*^ Homozygous ATM deletion

**Table 2:** Preclinical trials of PARPi and ATR-CHK1-WEE1 inhibitors in cancer cell lines

Histological type	PARPi resistance	Cell line	Mutations	Cell cycle checkpoint inhibitors + PARPi	Synergism	Effect of combo on ATR/ CHK1	Effect of combo on DSBs	Effect of combo on HR	Effect of combo on replication forks	Cell cycle alterations	Effect of combo on apoptosis	References
Ovary	Acquired PR	PEO1-PR	BRCA2-MUT	ATRi + PARPi (AZD6738 + Olaparib)	Yes	↑	↑	↓	↓	No	↑	[125]
		JHOS4-PR	BRCA1-MUT	ATRi + PARPi (AZD6738 + Olaparib)	Yes	↑	↑	No	↓	No	↑	[125]
		UWB1.289 SYR1	BRCA1-MUT	ATRi + PARPi (VE-821 + Olaparib)	Ye	–	–	↓	↓	–		[124]
		UWB1.289 SYR12*	BRCA1-MUT	CHK1i + PARPi (Prexasertib + Olaparib)	Yes	–		–	–	–		[86]
		UWB1.289 SYR13	BRCA1-MUT	ATRi + PARPi (VE-821 + Olaparib)	Yes	–	–	↓		–	–	[124]
		UWB1.289 SYR1	BRCA1-MUT	CHK1i + PARPi (Prexasertib + Olaparib)	Yes	–	–	–	–	–	–	[86]
		BR5-R1	BRCA1-MU	ATRi + PARPi (VE-821 + Olaparib)	Yes	–		–	–		–	[124]
	De novo PR	UWB1.289/BRCA1 ±	BRCA1 ±	ATRi + PARPi (VE-821 + Olaparib)	No	–	–	↓	No	–	–	[124]
				ATRi + PARPi (AZD6738 + Olaparib)	Yes	↑	↑	↓	–	No	–	[125]
		UWB1.289/53BP1−/−	BRCA1-MUT	ATRi + PARPi (AZD6738 + Olaparib)	Ye	–	–	–	–	No	–	[125]
		COV362/53BP1−/−	BRCA1-MUT	CHK1i + PARPi (Prexasertib + Olaparib)	Yes	–	–	↓	–	–	–	[86]
		Kuramochi	BRCA2-nonse mutation (c.6952C > T)	ATRi + PARPi (AZD6738 + Olaparib)	Yes	↑	↑	No	–	No	–	[125]
		PEO4	BRCA2-reversion mutation	ATRi + PARPi (AZD6738 + Olaparib)	Yes	↓	–	–	–	G2/M↑	↑	[141]
				CHK1i + PARPi (Prexasertib + Olaparib)	Yes	pATR↑/pCHK1↓	↑	↓	–	G2/M↓	↑	[143]
	Preexisting PR	OV-9	TP53-MUT BRCA-WT	CHK1i + PARPi (Prexasertib + Olaparib)	Yes	pATR↑/pCHK1↓	↑	↓	–	–	↑	[143]
				ATRi + PARPi (AZD6738 + Olaparib	Yes	↑	↑	–	–	–	↑	[123]
				CHK1i + PARPi (MK8776 + Olaparib)	Yes	↑	↑	–	–	–		[123]
		SKOV3	BRCA-WT	ATRi + PARPi + siBRCA1 (VE-821 + Velaparib + siBRCA1)	Yes	–	–	↓	–	–	–	[158]
				ATRi + PARPi (AZD6738 + Olaparib)	Yes	–	↑	–	–	–	↑	[123]
				CHK1i + PARPi (MK8776 + Olaparib)	Yes	–	↑	–	–	–	↑	[123]
		TOV112D	BRCA1-WT	CHK1i + PARPi (Prexasertib + Olaparib)	Yes	–	–	–	–	–	–	[86]
		ES2	BRCA1-WT	CHK1i + PARPi (Prexasertib + Olaparib)	Yes	–	–	–	–	–	–	[86]
		OVCAR-8	BRCA-WT	ATRi + PARPi + siBRCA1 (VE-821 + Velaparib + siBRCA1)	Yes	–	–		–	–	–	[158]
		OVCAR3	BRCA wild type; TP53-MUT(R248Q)	CHK1i + PARPi (Prexasertib + Olaparib)	Yes	pATR↑/Pchk1↓	↑	↓	–	G2/M↓	↑	[143]
				WEE1i + PARPi (AZD1775 + Olaparib)	Yes	–	–	–	–	M↑	↑	[159]
	PARPi-sensitive	PEO1	BRCA2-MUT(c.C4965G)	ATRi + PARPi (AZD6738 + Olaparib)	Yes	↓	↑	–	–	G2/M↓	↑	[141]
				ATRi + PARPi (VE-821 + Velaparib)	Yes	–	–	–	–	–	–	[158]
				CHK1i + PARPi (MK8776 + Olaparib	Yes	↓	↑	–	–	G2/M↓	↑	[141] [123]
				CHK1i + PARPi (Prexasertib + Olaparib)	Yes	pATR↑/Pchk1↓	↑	↓	–	G2/M↓	↑	[143]
	–	EM9	XRCC1−/−	ATRi + PARPi (NU6027 + Rucaparib)	Yes	–	–	–	–	–	–	[160]
Breast	Preexisting PR	HCC1937	BRCA1-deficient	ATRi + PARPi (VE-821 + Velaparib)	Yes	–	–	↓	–	–	–	[124]
	–	MCF7	–	ATRi + PARPi (NU6027 + Rucaparib)	Yes	–	↑	↓	–	–	–	[160]
Pancreas	Acquired PR	R-AKC	ATM-MUT	ATRi + PARPi + DNA-PKi + P-gpi (VE-822 + Olaparib + CC-115 + Elacridar)	Yes	–	–	–	–	G2/M↑	–	[132]
Lung	De novo PR	VC8-B2	BRCA2 + / +	ATRi + PARPi (NU6027 + Rucaparib)	Yes	–	–	↓	–	–	–	[160]
				CHK1i + PARPi (PF-47736 + Rucaparib)	Yes	↓	↑	↓	–	S↓G2/M↓ sub-G1↑	–	[144]
Endometrium	Preexisting PR	Hec50	BRCA-WT;TP53-MUT(intron 6)	BRCA-WT;TP53-MUT(intron 6)	Yes	–	–	–	–	M↑	↑	[159]
Gastric	–	MKN45	–	WEE1i + PARPi (AZD1775 + Olaparib)	Yes	–	↑	↓	–	G2/M↓	↑	[146]
	–	AGS	–	WEE1i + PARPi (AZD1775 + Olaparib)	Yes	–	↑	↓	–	G2/M↓	↑	[146]
Brain	–	MGG18	Non MYC-MUT	ATRi + PARPi (VE-822 + Olaparib)	Yes	↑	↑	↓	–	–	↑	[161]

*PARPi* PARP inhibitors; *Combo* Combination; *Acquired PR* Acquired PARP inhibitors resistance; De novo* PR* De novo PARP inhibitors resistance; *Preexisting PR* Preexisting PARP inhibitors resistance; *BRCA2-MUT* BRCA2-mutant; *BRCA1-MUT* BRCA1-mutant; *TP53-MUT* TP53 mutant; *ATM-MUT* ATM mutant; *ATRi* ATR inhibitors; *CHK1i* CHK1 inhibitors; *WEE1i* WEE1 inhibitors; *DNA-Pki* DNA-PK inhibitors; *P-gpi* P-gp inhibitors; *pATR* Phosphorylated ATR; *pCHK1* Phosphorylated CHK1

### *ATM/CHK2/p53 alterations* + *PARPi*

#### The combined blockade of ATM and PARP activity induces synthetic lethality to some extent

ATM is a member of the serine/threonine protein kinase family that participates in phosphorylation at cell cycle checkpoints, repair of DSBs, and processes in senescence and apoptosis. ATM mutations have been estimated to be present in 6.7% of castration-resistant prostate cancer (CRPC) and 12% of sporadic PDAC patients [162, 163]. The rate of ATM loss has been reported to be approximately 11% in CRPC patients. ATM alterations have been associated with elevated genomic instability in CRPC and PDAC patients. Whole-exome sequencing (WES) identified that the HRD burden, such as that caused by telomeric allelic imbalance (NtAI, *p* = 0.005) and large-scale transitions (LSTs, *p* = 0.048), was correlated with ATM loss in CRPC biopsy samples [162]. Additionally, the number of chromosomal aberrations is high, the aneuploidy and anaphase defects, the extent of structural rearrangements of chromosome 7 and chromosome *Y*, and even the degree of chromothripsis are frequent in ATM-deficient AKC cell lines, characteristic similarly to those in human PDAC [163]. Another study was dedicated to assessing the HRD score of a prostate cancer cohort composed on the basis of the extent of heterozygosity loss, NtAI and LSTs. The HRD score is used to evaluate the level of HR deficiency in clinic. Although evidence of chromosomal instability has been previously shown, as mentioned above in this study, the HRD score of germline ATM mutations was lower than that of germline BRCA2 mutations (16.5 vs. 27) [164]. A series of preclinical research studies have also indicated that cancer cell with ATM deficiency was less sensitive to PARPi than that with BRCA2 mutations. In ATM-deficient prostate and pancreatic cancer cells treated with a PARP inhibitor (rucaparib), lower level but not abrogated HRR was observed. This finding was confirmed by a reduction in the number of RAD51 foci, an increase in γH2AX and 53BP1 foci, a decrease in the number of GFP+ cells. The HRR rate that was the same as that of ATM-wild-type cells treated with ATM inhibitors. Consistent with this result, combination therapy with PARPi, ATM blockade, plus ATR inhibitor may be used to abolish HR [162]. Moreover, a triplex regimen of PARPi, ATRi, and DNA-PKi demonstrated a better synergistic effect in ATM-null PDAC cell lines [165]. In addition to the collaborative impairment caused by DSBs, DNA fiber assays showed that ATM knockout seemed to stimulate an alternative DNA damage bypass, which may have been targeted by PARPi [163]. Concerning the resistance mechanisms of PARPi in ATM-deficient cell lines, the overexpression of MDR genes, including Abcb1 (MDR1) and Abcg2 (Brcp), and the tendency for the epithelial-to-mesenchymal transition (EMT) were identified as primary genetic alterations. Overall, ATM alterations eroded genome stability and increased the number of more DSBs to a certain extent, providing synergistic exploitability with PARPi. Moreover, evidence shows that multidrug combinations that target PARP, ATR, ATM, and DNA-PK require further investigation to yield better synthetic lethality.

#### Targeting CHK2 efficiently lowers the hematological toxicity caused by PARPi

Accumulated evidence shows that PARP2 plays an important role in sustaining hematopoiesis, especially in erythropoiesis [166, 167]. Under steady-state conditions or stress conditions, PARP2 is closely related to maintaining the survival of hematopoietic stem/progenitor cells (HSPC) by facilitating the DNA repair progression and inhibiting p53/Puma-dependent apoptosis [166]. Besides, PARP2 sustains the life span of erythrocytes and participates in the differentiation process of erythroid progenitors by limiting replication stress. The mice loss of PARP2 in the long term were observed in bone marrow failure and chronic anemia [167]. Apart from PARP2’functions in hematology, PARP1 and PARP2 are jointly cooperative in sustaining T cell or B cell homeostasis in immune response [168, 169]. Given that the available PARP inhibitors in clinic primarily target both PARP1 and PARP2 proteins, the administration of PARPi inevitably leads to hematological adverse events and immune dysfunctions [10, 169]. Nowadays, it has been proved that selectively inhibiting PARP1 could bypass such potential side effects [170, 171]. Moreover, co-targeting CHK2-PARPs could also reduce hematological toxic events resulting from PARPi therapy.

CHK2, a molecule downstream of ATM, is a serine/threonine kinase that phosphorylates CDC25, BRCA1, and p53 and primarily participates in stopping the G1/S transition during cell cycle arrest, DNA damage signaling, and apoptosis. An ATP-competitive inhibitor of CHK2, CCT241533, has been found to synergize with PARPi in several tumor cell lines. PARP inhibitor treatment has been shown to activate CHK2 through autophosphorylation at Ser516, and CHK2i impaired activated CHK2, leading to an increased apoptosis rate [172]. In preclinical models, the administration of PARPi led to hematological toxicity, especially reducing the numbers of reticulocytes, immature CD4+CD8+ thymocytes, pro-B/pre-B cells, and immature B cells. Consistent with these findings, anemia (18% grade 3) and neutropenia (4% grade 3) were identified as adverse effects detected through the analysis of the blood of patients who had received PARPi in a clinical setting. The cytotoxicity of PARPi has been associated with p53 Ser23 phosphorylation, which is blocked by CHK2 inactivation. Therefore, targeting CHK2 (BML-277, also binding to the ATP pocket) blunted PARP inhibitor toxicity in p53-wild-type pro-B/pre-B cells but not in p53-deficient ovarian cancer cells, indicating that CHK2 inhibition may be a promising chemoprotective mechanism that can be leveraged to maintain olaparib efficacy [173]. In addition to chemoprotective function, CHK2 inhibitor, compound 2 h (BML-277), shielded CD4+ and CD8+ T cells from the effects of ionizing therapy [174]. In general, the inhibition of CHK2 potentiates the killing effect of PARPi in certain tumor cell lines but lowers the toxicity of PARPi in certain premature blood cells [172, 175].

#### *TP53 in different forms of tumors shows differential synergistic effects with PARPi mediated *via* distinct mechanisms*

TP53 is a tumor suppressor gene that participates in many cellular biological processes and is involved in the DDR, apoptosis, and oxidative stress responses [176, 177]. Mutations in p53 occur at an estimated rate of 80% in TNBC and more than 96% in HGSOC [173, 176]. However, in colorectal cancer (CRC), wild-type TP53 was a prerequisite for a PARP inhibitor response because PARPi targeted molecules downstream of p53, especially, GDF15, PLK2, MDM2, TP53INP1, and RRM2B, and attenuated RAD51 foci formation, but not the HRD score [178]. Additionally, through targeted blocking of the long noncoding RNA RMRP, an inhibitor of TP53, olaparib may completely reactivate TP53 function to drive apoptosis and ferroptosis [138]. Similarly, in breast cancer and glioblastomas, wild-type TP53 and the TP53-R273H gain-of-function mutant mediated the shuttling of BRCA1 from the nucleus to the cytosol, resulting in impaired HRR capacity and yielding a better response to PARPi after IR pretreatment [179]. Moreover, some gain-of-function mutations (R273H and R248W) in TNBC model cells led to increased PARP abundance on nascent DNA strands, which was synergized via the action of talazoparib through an enhanced PARP trapping mechanism [176]. However, in TP53 loss-of-function mutant bladder cancer cells, neither the remaining capacity in regulated HR function nor cell cycle disturbance mediated through p21 was the cause of cell sensitivity to olaparib. In fact, the loss-of-function mutation of TP53 activated oxidative stress pathways and the overexpression of PARP1, which drove cell responsiveness to PARPi and increased their sensitivity to radiation therapy [177]. Taken together, wild-type and mutant TP53 conferred sensitivity to PARPi in an array of tumors through various mechanisms.

### *Targeting ATR/CHK1/WEE1* + *PARPi: preclinical data*

#### Cotreatment with PARPi and ATRi exerts a primarily synergetic effect on cell cycle arrest, HR impairment, and replication fork collapse, resulting in tumor eradication

In cancer cells, common TP53 mutations usually lead to the loss of function at the G1 cell cycle checkpoint, resulting in increased cell reliance on the S and G2/M checkpoints to repair DNA damage [95]. ATR is the main regulator of the DDR and RS response and can protect the G2/M cell cycle checkpoint and replication forks through its interaction with PARP. Preclinical trials to determine whether a combination therapy consisting of PARPi and ATRi exerts a synergistic effect across a number of cancer types are needed. Notably, elevated baseline levels of activated ATR and CHK1 have been detected in several PARP-inhibitor-sensitive ovarian cancer cells, indicating a high level of preexisting RS that made these cells highly dependent on the ATR/CHK1 pathway with increased sensitivity to ATR inhibitor therapy [150, 180]. Consistent with this finding, PARP inhibitor monotherapy, indeed, caused DSB formation, which relies on delayed G2 for repair. Although, treatment using ATRi released G2 arrest and induced aberrant chromatid breaks, premature gaps in the M phase leading to mitotic catastrophe. This is the main reason that single-agent PARPi suppressed only tumor growth but the combination of PARPi and ATRi eliminated the tumor burden [180]. Collectively, cotreatment using ATR inhibitors and PARPi revealed evidence of synthetic lethality in many types of tumor cells, and previous explorations have undoubtedly paved the way for further investigation into the spectrum of PARP-inhibitor-resistant cells.

Combination therapy using ATRi and PARPi has been proven to exert a synergistic effect in an array of PARP-inhibitor-resistant cancer cells. For instance, ATRi preferentially resensitized a series of PARP-inhibitor-resistant HGSOC cell lines with BRCA1/BRCA2 deficiency to PARPi [149, 180]. Consistent with this finding, the overexpression of ATR has also been observed in PARP-inhibitor-resistant cells. During cotreatment with PARPi and ATRi, the modulation of the G2/M cell cycle checkpoints was blunted [150, 180]. In this study, individual ovarian tumor cells revealed the heterogeneity involved in resistance mechanisms, rewiring the HRR pathway, regaining replication fork capacity and protection, and the upregulation of drug efflux pump MDR genes independent of BRCA1/BRCA2 levels. By bypassing BRCA1, ATR participates in the restoration of HR by phosphorylating RPA and recruiting other substrates, namely PALB2 and BRCA2, to DSBs. Thus, ATRi disrupted the rewired HR pathways by impairing the p-RPA/PALBC2/BRCA2 signaling pathway and overcame a typical PARP inhibitor resistance mechanism [149, 150]. Additionally, the combined regimen consisting of PARPi and ATRi also showed a synergistic effect in stalling replication forks, slowing fork speed and causing fork asymmetry [149, 150, 180]. ATRi were observed to regulate XRCC3, a paralog and regulator of RAD51 filaments, inhibiting RAD51 dwelling at stalled replication forks in vitro and in vivo [149].

#### CHK1 blockade sensitizes BRCA-WT cancer cells to PARPi by impairing HRR

CHK1 inhibitor monotherapy or combinations of CHK1i with PARPi can be used to modulate cell cycle distribution and DNA repair. CHK1’s protection of replication forks has shown promise for dissecting the connection between DDR and RS response pathways. In addition to participating in G2/M cell cycle arrest, CHK1 is involved in the repair of DSBs through the facilitation of BRCA2 (C-terminal domain)-Rad51 (T309) phosphorylation, which is associated with the transnuclear localization of the RAD51 foci [181]. CHK1 inhibitors (CHK1i, MK8776, LY2606368, and PF-477736) have demonstrated cytotoxic effects in BRCA-wild-type, BRCA-mutant, and even PARP-inhibitor-resistant ovarian and lung cancers [105, 180, 182]. As illustrated above, the administration of PARPi increased DNA damage in cancer cells and made them more reliant on activated pATR/pCHK1 for survival. Prexasertib (LY2606368) is a potent CHK1 inhibitor and, to a lesser extent, a CHK2 inhibitor. Its use as a monotherapy has led to elevated levels of pKAP1 and *γ*-H2AX, which are markers of DDR, and pRPA32, a marker of RS in HGSOC models in vivo and 14 PDX models in vitro [105, 181]. In contrast to cotreatment with PARPi and ATRi, the PARP-CHK1 inhibitor (such as olaparib-MK8776) combination was effective only for tumor suppression and did not eliminate cancer cells. In PARP-inhibitor-treated cells, blocking CHK1 released the G2/M cell cycle checkpoint, and cells with unrepaired DSBs prematurely entered mitosis, resulting in the accumulation of chromosome breaks and even mitotic catastrophe [180]. Interestingly, the CHK1 inhibitor (PF-477736) potentiated the cytotoxicity of APRPi only in the BRCA2-reversion mutant V-C8 B2 cells but not BRCA2-mutant V-C8 cells. The distinct sensitivity toward the HRR abolishment was caused by PF-47736 in V-C8 B2 cells, as was demonstrated by completely impaired RAD51 foci formation and HRD phenotype acquisition after PARP inhibitor treatment [183]. Additionally, cotreatment using prexasertib and olaparib synergistically reduced HR capacity and reversed the stability of replication forks across a spectrum of HGSOC cell lines, including de novo PARP-inhibitor-resistant COV362 cells and acquired PARP-inhibitor-resistant UWB1.289 SYR12 and SYR14 cells. Furthermore, TOV112D and ES2 cells demonstrated characteristics of the clear cell or endometrioid subtypes of ovarian tumors, which responded poorly to standard therapy regimens but showed sensitivity to a combination of prexasertib and olaparib [105].

#### A sequential therapy regimen comprising PARPi with WEE1 inhibitors (WEE1i) preserves the curative effect and diminishes side effects

Consistent with the roles of ATR/CHK1, WEE1 is a kinase that commonly expresses during RS response and provides permission for entry into mitosis by phosphorylating and inactivating CDK2 and CDK1, two cell cycle-dependent kinases found at the G1 and G2/M transition points, respectively [184]. Combination treatment using PARPi and WEE1i demonstrated a synergistic effect across a spectrum of tumor types, including endometrial and ovarian cancer, small-cell lung cancer (SCLC), non-small-cell lung cancer (NSCLC), and gastric cancer [181, 184]. Inventive patient circulating tumor cell-derived explant (CDX) models of SCLC mimicked chemosensitive, chemoresistant, and treatment-naïve patient characteristics and showed that cotreatment with PARPi and WEE1i showed superior efficacy compared with monotherapy [184]. Similar to the combination of PARPi and ATR/CHK1 blockade, PARPi with WEE1i demonstrated similar synthetic lethality in vitro and in vivo in two gastric cancer cell lines, as is evident from an increase in the number of DSBs, impaired HRR or HR deficiency, disrupted cell cycle arrest, and an increase in the apoptosis rate [185]. PARP inhibitor treatment induced G2 arrest to gain time for DDR, while cotreatment using WEE1i in PARP-inhibitor-treated cells released the cells from G2 arrest and caused them to prematurely enter the M phase by abrogating two core mitotic gatekeepers, cdc2Y15 and FOXM1, leading to a mitotic catastrophe [181]. More importantly, another preclinical study revealed that the synergistic lethality caused by a combination of talazoparib and adavosertib was highly schedule-dependent. In tumor cells, the sequential administration of PARPi and WEE1i retained curative effects through each round and induced minimal cytotoxicity. In normal cells, this sequentially applied regimen ameliorated toxicity by inducing low levels of RS, decreasing DNA damage, reversibly arresting the G1 cell cycle checkpoint to ensure cell survival [181]. Although for the majority of tumors, p53 mutations abrogate the G1/S cell cycle checkpoint and cancer cells become increasingly reliant on G2/M arrest in which WEE1 exerts an important effect. However, we cannot rule out p53 mutations as predictive biomarkers of WEE1 inhibitor sensitivity [184]. Baseline endogenous RS, excessive oncogenic stress (such as CCNE amplification), or RS induced by therapy in tumor cells but not in normal cells was a determinant for the stratification of patients who can benefit from sequential treatment with PARPi and WEE1i [181, 184]. In NSCLC, the combination of PARPi and WEE1i induced DNA RS and potentiated sensitivity to radiation by inducing PARP1 trapping and nucleotide consumption [186]. Collectively, the sequential administration of PARPi with WEE1i shows a better response than simultaneous administration. The elevated RS level exhibited a predictive response for this combination.

## PARP inhibitor combination strategies with cell cycle checkpoint inhibitors: clinical trials

### *ATM/CHK2/p53 mutations* + *PARPi*

**Fig. 5:**
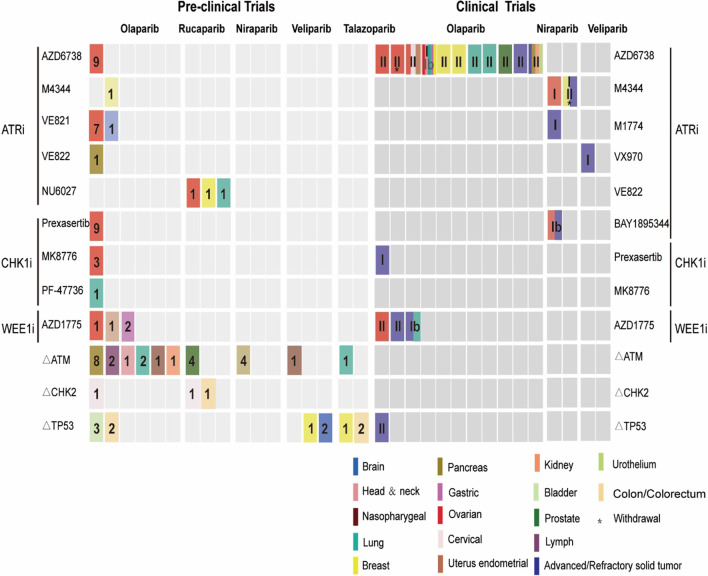
Preclinical and clinical trials targeting cell cycle checkpoints and PARP inhibitors (PARPi). The different colors of the blocks represent various cancer tissues, labeled with details. On the left side, the number in each block indicates the number of cell lines treated with the indicated combination. Each block on the right indicates a clinical trial, and the trial phase is indicated with Roman letters. *ATRi* ATR inhibitors; *CHK1i* CHK1 inhibitors; *WEE1i* WEE1 inhibitors; △*ATM* ATM alterations; △*CHK2* CHK2 alterations; △*TP53* TP53 alterations

A few clinical trials that have focused on the combination of PARPi and ATM/CHK2/p53 alterations are being conducted. A phase III clinical trial, PROfound (NCT02987543), which was conducted with advanced prostate tumors, showed promising results in patients harboring BRCA1, BRCA2, or ATM alterations (Cohort A) with prolonged radiographic progression-free survival (rPFS) (median 7.4 months vs. 3.6 months; the hazard ratio for progression or death was 0.34; 95% CI of 0.25–0.47) and elevated overall survival (OS) (18.5 months vs. 15.1 months; hazard ratio for death was 0.64; 95% CI of 0.43–0.97) compared with the control. However, a separate analysis conducted on tumors with ATM mutations showed a disappointing outcome (the hazard ratio for rPFS was 1.04; 95% CI of 0.64–1.87) [187, 188]. In PROfound study, participants with CHK2 mutations (Cohort B) showed limited sensitivity to olaparib [187]. A phase II TRITON2 study (NCT02952534) categorized patients with metastatic CRPC (mCRPC) into ATM, CDK12, CHK2, and other DDR gene groups to determine whether non-BRCA mutations produced a response to PARPi. Although a limited benefit was observed with a PSA response and radiographic response for the ATM cohort [2/19 (10.5%) and 2/49 (4.1%), respectively] and the CHK2 cohort [1/9 (11.1%) and 2/12 (16.7%), respectively], most patients with ATM mutations acquired disease stabilization after treatment with rucaparib [189]. Similarly, stable disease of multiple durations was also found in the TALAPRO-1 study (NCT03148795) conducted for talazoparib with mCRPC patients harboring ATM mutations (*n* = 17) or CHEK2 alterations (*n* = 9) [190]. In TBCRC 048 (NCT03344965) study, a phase II study that evaluated the efficacy of olaparib in metastatic breast cancer patients, inferior results of no response were observed in eligible participants with ATM- or CHK2-only mutations [191]. Above all, the combination of ATM/CHK2/p53 mutations and PARPi need to be further explored through clinical trials, especially considering the limited benefits of these therapy regimens. Detailed information related to preclinical and clinical trials for evaluating cell cycle checkpoint inhibitors with PARPi is shown in Fig. 5 and Tables 1, 2, and 3.

**Table 3:** Clinical trials of PARPi and cell cycle inhibitor administration in cancers

NCT number	Intervention	Target	Disease setting	Phase	Trial status	Result
04,149,145	M4344 + Niraparib	ATRi + PARPi	PARPi-resistant recurrent ovarian cancer	I	Not yet recruiting	/
04,655,183	M4344 + Niraparib	ATRi + PARPi	Advanced solid tumors Breast cancer with DDR mutations	I/II	Withdrawn	/
04,170,153	M1774 + Niraparib	ATRi + PARPi	Metastatic or locally advanced Unresectable solid tumors	I	Recruiting	/
02,723,864	VX-970 + Veliparib + Cisplatin	ATRi + PARPi + Platinum	Refractory solid tumors	I	Completed	0/3;0/3;2/6;0/7;0/3;3/25;0/6 ORRs in each arms 1/3;2/3;5/6;4/7;3/3;10/25;3/6 DCRs in each arms
04,267,939	BAY1895344 + Niraparib	ATRi + PARPi	Advanced solid tumors (excluding prostate cancer) Ovarian cancer	Ib	Recruiting	/
02,264,678	AZD6738 + Olaparib	ATRi + PARPi	Advanced solid malignance—H&N SCC, ATM Pro/Def NSCLC, gastric, breast and ovarian cancer	I/II	Recruiting	/
03,462,342	AZD6738 + Olaparib	ATRi + PARPi	Recurrent ovarian cancer (platinum-sensitive or platinum-resistant cohort)	II	Recruiting	/
04,065,269	AZD6738 + Olaparib	ATRi + PARPi	Relapsed ovarian (fallopian tube/primary peritoneal) and endometrial (uterus) clear cell carcinomas with/without loss of ARID1A expression Other rare relapsed gynecological cancers (endometrioid ovarian carcinoma, endometrioid endometrial carcinoma, cervical adenocarcinoma, cervical squamous, ovarian carcinosarcoma and endometrial carcinosarcoma) irrespective of ARID1A status	II	Recruiting	/
03,682,289	AZD6738 + Olaparib	ATRi + PARPi	Clear cell/metastatic/stage III/IV renal cell carcinoma Locally advanced/metastatic/stage III/IV pancreatic cancer Locally advanced/metastatic malignant solid neoplasm Metastatic urothelial carcinoma	II	Recruiting	/
04,239,014	AZD6738 + Olaparib	ATRi + PARPi	Ovarian cancer	II	Withdrawn	/
04,090,567	AZD6738 + Olaparib	ATRi + PARPi	Advanced or metastatic breast cancer with germline BRCA1/2 mutations	II	Recruiting	/
02,937,818	AZD6738 + Olaparib	ATRi + PARPi	Platinum refractory extensive-stage small-cell lung carcinoma	II	Active, not recruiting	/
03,787,680	AZD6738 + Olaparib	ATRi + PARPi	Metastatic castration-resistant prostate cancer (mCRPC) with DNA repair proficiency (DRPro) mCRPC with DNA repair deficiency (DRDef)	II	Active, not recruiting	/
03,428,607	AZD6738 + Olaparib	ATRi + PARPi	Relapsed small-cell lung cancer (SCLC)	II	Completed	/
03,330,847	AZD6738 + Olaparib AZD1775 + Olaparib	ATRi + PARPi WEE1i + PARPi	Metastatic triple-negative breast cancer(TNBC) with BRC1/2 mutations Metastatic TNBC with other HRR gene mutations other than BRCA1/2 Metastatic TNBC without any mutation of HRR genes	II	Active, not recruiting	/
02,576,444	AZD6738 + Olaparib AZD1775 + Olaparib	ATRi + PARPi WEE1i + PARPi	Advanced solid tumors with mutations in homologous—DNA repair (HDR) genes or mutations such as ATM, CHK2, MRN (MRE11/NBS1/RAD50), CDKN2A/B and APOBEC Advanced solid tumors with mutations of TP53 or KRAS gene	II	Active, Not Recruiting	/
03,057,145	Prexasertib + Olaparib	CHK1i + PARPi	Advanced solid tumors	I	Completed	/
02,511,795	AZD1775 + Olaparib	WEE1i + PARPi	Refractory solid tumors Relapsed small-cell lung cancer (SCLC)	Ib	Completed	11.1% ORR in the total polulation; 55.7% DCR in the total population
03,579,316	AZD1775 + Olaparib	WEE1i + PARPi	PARPi-resistant recurrent ovarian (fallopian tube/primary peritoneal) cancers	II	Recruiting	/

### *ATR/CHK1/WEE1 inhibitors* + *PARPi*

A phase I/II, open-label, multicenter clinical trial (NCT02264678), module 2 of part B5 of an expansion study was reviewed. Ovarian cancer patients who were platinum-sensitive had previously been presented with progression despite treatment with PARPi and harbored either germline, or somatic BRCA mutations were recruited to receive combined AZD6738 and olaparib treatment [192]. The initial antitumor data on the pharmacodynamics for this trial were published in 2018. One patient acquired a response evaluation criteria in solid tumors (RECIST)-identified complete response (CR), and 6 patients showed a partial response (PR), with one outcome unconfirmed among a total of 39 patients suffering from advanced solid tumors, including ovarian cancer. Regarding side effects, dose-limiting toxicity (DLT)-related thrombocytopenia and neutropenia along with other side effects such as anemia, fatigue, and several familiar entera and respiratory symptoms, were found in more than one-fifth of the patients (with ≥ G3 events). According to the trial assessing AZD6738 along with the continuous use of olaparib tablets, a comparable proper phase 2 dose of AZD6738 at 160 mg od from Day 1 through 7 plus olaparib 300 mg bd (in a 28-day cycle) was recommended. Importantly, a preliminary pharmacokinetic (PK) evaluation was also conducted with AZD6738, and further study is warranted before translation of this combination from bench to bedside [193]. Collectively, 5 phase 1 or phase 2 clinical trials are currently being conducted on combination treatments of olaparib and AZD6738, which have been shown to be promising and warrant further investigation. Notably, most of these studies are in the recruitment stage, as indicated in Table 2. Additionally, M6620, also known as VX-970, was the first potent ATR inhibitor to have been applied to clinical trials. M6620 has the ability to slow down the growth of tumors, which has been confirmed in an array of preclinical trials. An ongoing phase 1 study (NCT02723864) of M6620 plus veliparib and cisplatin in patients with refractory solid malignancies (Table 2) is aimed at assessing whether veliparib and M6620 can induce a “BRCAness”-like phenotype to augment sensitivity to cisplatin toxicity. A statement was released indicating that a PR was observed in 3 of the 22 patients, including 1 patient with BRCA-proficient ovarian cancer, and 12 of the 22 patients did not show any sign of progression [162]. Overall, several hematological toxicities, including hypophosphatemia, thrombocytopenia, and leucopenia lymphopenia, have been reported [192]. This clinical study will hopefully lead to the identification of a suitable method for treatment that does not induce adverse side effects in ovarian cancer patients.

Prexasertib is a bioavailable CHK1 inhibitor that has recently entered phase 1 clinical trials. In addition, combination therapy using prexasertib and PARPi has been evaluated in solid tumors (NCT03057145). Collectively, twenty-nine participants were enrolled in this study, and eighteen of these patients were PARP-inhibitor-resistant HGSOC patients with BRCA1 mutations. Notably, four BRCA1-mutant PARP-inhibitor-resistant patients acquired a PR. An increase in the number of DSBs and diminished HR capacity have been proven with tumor biopsy samples with RAD51 foci reduction and accumulation of DNA damage markers, such as γH2AX, pKAP1, and pRPA. The side effects of combination treatment were primarily hematological toxicity (leukopenia (83%), neutropenia (86%), thrombocytopenia (66%), and anemia (72%)). Based on this result, the maximum tolerated dose (MTD)/recommended phase 2 dose (RP2D) of prexasertib was 70 mg/m^3^ intravenously administered in addition to 100 mg of olaparib administered orally twice per day.

A phase Ib study sponsored by Astra Zeneca (NCT02511795) (Table 2) evaluating the coadministration of a highly selective WEE1 inhibitor, AZD1775, and the potent PARP inhibitor olaparib was conducted with 119 patients with refractory solid tumors, including 26 ovarian cancer patients, and was completed on October 16, 2019 [192, 194]. The synergistic antitumor capacity of adavosertib (AZD1775) combined with olaparib was observed in patients irrespective of BRCA mutation status, with a preliminary overall response rate (ORR) of 11% and a PFS of 3.2 months overall. Moreover, nearly all toxic and other side effects such as anemia, neutropenia, and thrombocytopenia, were generally manageable. Almost important, this study identified a MTD/RP2D of 175 mg of adavosertib BID (3/4) for 2/3 weeks plus 200 mg BID of olaparib, while the RP2D for the QD schedule was 200 mg of adavosertib (3/4) for 2/3 weeks plus 200 mg BID of olaparib, providing a basis suitable for conducting a phase II study [194]. Given that PARP inhibitor resistance can be acquired in patients with recurring ovarian cancer, a randomized, two-arm phase II trial is currently being conducted in which these patients are being treated with AZD1775 alone or in combination with olaparib, with the aim of blocking the progression of tumor cells [192]. Remarkably, tumors with a TP53 mutation displayed dysfunction of the G1/S checkpoint and were more reliant on the kinase activity of WEE1 to occlude G2/M cell cycle progression and provide enough time for DNA repair [195, 196]. Intriguingly, another phase II study (NCT02576444) applied cotreatment of AZD1775 plus olaparib to participants who harbored either TP53 or KRAS mutations to evaluate the ORR of tumors with specific molecular alterations [192].

## Conclusions and perspectives

Notably, evidence that has been accumulated over the past few years indicates that the DDR and RS response may contribute to protecting genome integrity and stability in cancer cells. The coordination exists in these two pathways. Furthermore, cell cycle checkpoints are the dominant components of the RS response. PARP1 regulates various DDR processes. They share and undergo interactions in response to DNA injuries and replication disruption, showing genetic vulnerabilities for developing combination strategies.

Overall, a more in-depth understanding of how the synergistic effects between cell cycle checkpoint inhibition and PARP1i are generated will contribute to a novel perspective and exploitability for the translation of these treatments from preclinical to clinical trials. Clearly, cancer cells exhibit defective G1/S checkpoints due to ATM/CHK2/TP53 mutations and are more reliant on G2/M checkpoints, stimulating the ATR/CHK1/WEE1 pathway. Additionally, DNA damage due to PARP inhibitor administration renders cancer cells more reliant on the ATR/CHK1/WEE1 pathway to complete the repair process. Remarkably, targeting cell cycle checkpoints and PARP1 results in the downregulation of replication fork-stabilizing factor genes and an increase in DSB formation resulting from fork collapse. Moreover, due to the inhibition of G2-M cell checkpoints, cells with unrepaired DSBs are inappropriately permitted to proceed into mitosis, leading to broken chromatid accumulation and mitotic catastrophe. Although PARPi have been approved to treat ovarian, breast, and pancreatic cancers, only the small HRD patient population benefits from PARP inhibitor monotherapy, which merely suppresses tumor growth. Emerging resistance to PARPi and toxic side effects are still important areas for exploration. Preliminary synergistic efficacy and safety have been observed in preclinical and clinical trials of cell cycle checkpoint-PARP inhibitor combinations. Therefore, further study of these combinations in various tumor settings is warranted to determine optimal treatment schedules and doses. Clearly, designing rigorous and structured clinical trials that involve distinct PARP inhibitor resistance mechanisms remains a challenge and is worthy of further exploration.

Moreover, factors remain to be resolved in more detail. One key issue is the lack of precise genetic markers for the accurate prediction of PARP inhibitor resistance or the stratification of patients who might benefit from these combination therapies. Additionally, the identification of biomarker levels and minimization of their cost remain to be realized. Another primary challenge that needs to be addressed is determining the manner in which the certain combination regime can yield distinct reactions in normal and cancerous cells, which is important for minimizing overlapping toxicity while maintaining drug efficacy, such as that achieved via sequential therapy using PARPi with WEE1i or by lowering doses. Besides, the emerging highly selective PARP1 inhibitor AZD5305, AZD9574 is about to refine hematological adverse events and immune dysfunction caused by first-generation PARP inhibitors. Moreover, this second-generation PARPi monotherapy or combinations with other chemotherapeutics retained evident tumor regression at a lower dose in the long term, indicating the potential reduction in PARPi-resistance [170, 171, 197]. Fortunately, AZD9574 has been proven to be penetrant in brain and may benefit HRD+ breast tumors with brain metastases in vivo [197]. The optimal scheduling of these combination therapies in which the therapeutic window available for the administration of tolerable synergistic cocktails for both primary cancers and PARP-inhibitor-resistant tumors remains to be elucidated. Of particular concern is the manner in which different genomic aberrations, racial disparities, and PARP inhibitor resistance heterogeneity may produce distinct responses to targeted combination treatment strategies. Correspondingly, the specific resistance mechanisms and individual defective targets need to be identified to provide personalized patient care and beneficial, cost-effective, and well-tolerated therapeutic strategies.

In conclusion, the primary necessity is a more rigorous, encompassing, dynamic understanding of interactions that modulate cell cycle checkpoints and PARP1 with respect to DDR and RS to further explore the future feasibility for novel combinational cancer therapies that exploit genome instability.

## Data Availability

Not applicable.

## Abbreviations

a-NHEJ: Alternative nonhomologous end joining
APC/C: The anaphase-promoting complex/cyclosome
ATM: Ataxia–telangiectasia mutated
ATR: Ataxia–telangiectasia mutated rad-3-related
ATRIP: ATR-interacting protein
BER: Base excision repair
BRG1: Brahma-related gene 1
CDKs: Cyclin-dependent kinases
CKIs: CDK inhibitors
CDK2: Cyclin-dependent kinase 2
CFSs: Late-replicating common fragile sites
CD: Co-directional
CGH: Comparative genomic hybridization
CtIP: CtBP-interacting protein
CIN: Chromosome instability in mitosis
CRPC: Castration-resistant prostate cancer
CRC: Colorectal cancer
CDX: Cell patient-derived explants
CR: Complete response
DDR: DNA damage response
DSBs: DNA double-strand breaks
dNTPs: Deoxyribonucleoside triphosphates
dNDPs: Deoxyribonucleotide diphosphates
DLTs: Dose-limiting toxicities
ERFSs: Early replicating fragile sites
EZH2: Enhancer of zeste homologue 2
EXO1: Exonuclease 1
EMT: Epithelial-to-mesenchymal transition
FA: The Fanconi anemia
HRD: Homologous recombination deficiency
HR: Homologous recombination
HU: Hydroxyurea
HO: Head-on
H3K4: Lys4 on histone 3
H3K27me3: Methylated Lys27 on histone 3
HGSOC: High-grade serous ovarian carcinoma
IR: Irradiation
LSTs: Large-scale transitions
MMR: Mismatch repair
MRE11: Meiotic recombination 11 homolog 1
MDR1: The multidrug resistance protein 1
mCRPC: Metastatic castration-resistant prostate cancer
MTD: The maximum tolerated dose
NER: Nucleotide excision repair
NDPs: Nucleotide diphosphates
NtAI: Telomeric allelic imbalance
NSCLC: Non-small-cell lung cancer
PARPi: Poly (ADP-ribose) polymerase inhibitors
PARG: Poly (ADP-ribose) glycohydrolase
PIKKs: Phosphatidylinositol 3-kinase-related protein kinases
PDAC: Pancreatic ductal adenocarcinoma
PR: Partial response
PK: Pharmacokinetic
RS: Replication stress
ROS: Reactive oxygen species
RNR: Ribonucleotide reductase
RPA: Replication protein A
RP2D: Recommended phase 2 dose
rPFS: Radiographic progression-free survival
SSB: Single strand breaks
ssDNA: Single-strand DNA
SSBR: Single-strand break repair
SCEs: Sister chromatid exchanges
SCLC: Small-cell lung cancer
TRCs: Transcription–replication conflict
TopBP1: DNA topoisomerase 2-binding protein 1
TNBC: Triple-negative breast cancer
UV: Ultraviolet
WGS: Whole-genome sequencing
WES: Whole-exome sequencing
XRCC1: X-ray repair cross-complementing protein 1
